# IL-1β-MyD88-mTOR Axis Promotes Immune-Protective IL-17A^+^Foxp3^+^ Cells During Mucosal Infection and Is Dysregulated With Aging

**DOI:** 10.3389/fimmu.2020.595936

**Published:** 2020-11-06

**Authors:** Natarajan Bhaskaran, Fady Faddoul, Andre Paes da Silva, Sangeetha Jayaraman, Elizabeth Schneider, Prerna Mamileti, Aaron Weinberg, Pushpa Pandiyan

**Affiliations:** ^1^ Department of Biological Sciences, Case Western Reserve University, Cleveland, OH, United States; ^2^ Advanced Education in General Dentistry, Case Western Reserve University, Cleveland, OH, United States; ^3^ Department of Periodontics, School of Dental Medicine, Case Western Reserve University, Cleveland, OH, United States; ^4^ Department of Pathology, School of Medicine, Case Western Reserve University, Cleveland, OH, United States

**Keywords:** T_reg_, Foxp3, T_reg_17, IL-1β, *Candida*, fungal infection, senescence, aging

## Abstract

CD4^+^Foxp3^+^T_regs_ maintain immune homeostasis, but distinct mechanisms underlying their functional heterogeneity during infections are driven by specific cytokine milieu. Here we show that MyD88 deletion in Foxp3^+^ cells altered their function and resulted in increased fungal burden and immunopathology during oral *Candida albicans* (CA) challenge. Excessive inflammation due to the absence of MyD88 in T_regs_ coincided with a reduction of the unique population of IL-17A expressing Foxp3^+^ cells (T_reg_17) and an increase in dysfunctional IFN-γ^+^/Foxp3^+^ cells (T_reg_IFN-γ) in infected mice. Failure of MyD88^-/-^ T_regs_ to regulate effector CD4^+^ T cell functions correlated with heightened levels of IFN-γ in CD4^+^ T cells, as well as increased infiltration of inflammatory monocytes and neutrophils in oral mucosa *in vivo*. Mechanistically, IL-1β/MyD88 signaling was required for the activation of IRAK-4, Akt, and mTOR, which led to the induction and proliferation of T_reg_17 cells. In the absence of IL-1 receptor signaling, T_reg_17 cells were reduced, but IL-6-driven expansion of T_reg_IFN-γ cells was increased. This mechanism was physiologically relevant during *Candida* infection in aged mice, as they exhibited IL-1 receptor/MyD88 defect in Foxp3^+^ cells, loss of p-mTOR^high^T_reg_17 cells and reduced levels of IL-1β in oral mucosa, which coincided with persistent tongue inflammation. Concurrent with T_reg_ dysfunction, aging was associated with increased CD4^+^ T cell hyperactivation and heightened levels of IL-6 in mice and humans in oral mucosa *in vivo*. Taken together, our data identify IL-1β/MyD88/T_reg_ axis as a new component that modulates inflammatory responses in oral mucosa. Also, dysregulation of this axis in an aging immune system may skew host defense towards an immunopathological response in mucosal compartments.

## Introduction

CD4^+^CD25^+^Foxp3^+^ regulatory T cells (T_regs_) are central in controlling the magnitude of an immune response thereby regulating autoimmunity and maintaining mucosal tolerance (1). We and others have shown that 5%–10% of CD4^+^ T cells have a T_reg_ phenotype in normal oral mucosa (2–4). Molecular components that define their functional plasticity and heterogeneity are not completely characterized during mucosal infections, and appear to be driven by specific stimulation and cytokine milieu. *Candida* is an innocuous commensal in >60% of human population but causes opportunistic infections and chronic oral erythematous candidiasis in elderly individuals (5). Host pathogen recognition receptors including toll-like receptor (TLR)-2, Dectin, and EphA2 are known to recognize *Candida* (6, 7). C-type lectin receptor-Syk (spleen tyrosine kinase) adaptor CARD-9-IL-1β axis, IL-17 receptor signaling, and Th17 cells play important roles in antifungal immunity (8, 9). T_regs_ are critical for enhancing early Th17 host responses, as well as controlling excessive immunopathological responses during the resolving phase of oropharyngeal candidiasis (OPC). While thymic T_regs_ (tT_regs_) regulate systemic Th1 autoimmunity, peripheral T_regs_ (pT_regs_) are generated extrathymically at mucosal interfaces and control commensal microbiota composition and local inflammation (10, 11). Microbial stimulants are known to control pT_reg_ functions and the mechanisms have begun to be elucidated (12–14). Some studies imply that T_reg_ suppression can be bypassed by microbial signals such as toll-like receptor (TLR) ligands, myeloid differentiation primary response 88 (MyD88) signals, and pro-inflammatory cytokines (15–17). Others conclude that MyD88 and cMAF dependent microbial sensing by T_regs_ are shown to enhance their suppressive capacities (2, 18–23). Thus, the intrinsic role of MyD88 in mucosal T_regs_ during an infection remains to be defined. Here we show that IL-1β/MyD88 principally promotes the induction and proliferation of RORγt^+^IL-17^+^Foxp3^+^ cells (T_reg_17) in an mTOR dependent manner during *Candida* challenge. These cells are required for optimal resolution of infection and inflammation. Absence of IL-1β signaling in Foxp3^+^ cells also leads to an IL-6 driven expansion of T_reg_IFN-γ cells, which appear to coincide with immunopathology. While RORγt expressing Foxp3^+^ cells have been implicated in playing diverse roles in intestinal inflammation (13, 24, 25), our results demonstrate their immune-protective functions and the contrasting roles of IL-1β and IL-6 in determining their plasticity and function during an oral mucosa infection. Our data also highlight an age dependent dysregulation of this mechanism due to an imbalance in these cytokines. Collectively, these results demonstrate that IL-1β/MyD88 signaling augments T_reg_ functions and modulates mucosal immunity and also provide new insights in to a mechanism underlying immune-dysfunction in human aging and mucosal infections.

## Materials and Methods

### Mouse Cells, Patients, Human PBMC, and Gingival Biopsies

Mouse experiments were performed at Case Western Reserve University (CWRU) under an approval from the CWRU Institutional Animal Care and Use Committee, and followed all guidelines and regulations. Some of the experiments were also done at NIAID, NIH in compliance with the NIAID Institutional Animal Care and Use Committee’s guidelines and under an approved protocol. Young (6-9 weeks of age) *Myd88^flox/flox^*, *Foxp3-YFP^cre^* transgenic mice, BALB/cJ, C57BL/6J, *Foxp3^GFP^* reporter, CD45.1 congenic mice, and *IL-1R^-/-^* mice, as well as aged (12–18 months of age) C57BL/6 mice were purchased from Jackson Laboratories. Animals of both genders were used for experiments. Foxp3 specific-MyD88 deficient mice (MFYcre) were generated by breeding *Myd88^flox/flox^* and *Foxp3-YFP^cre^* (FYcre) mice. Human PBMC, gingival biopsies and saliva were obtained under a protocol approved by the University Hospitals Cleveland Medical Center Institutional Review Board. Informed consents were obtained from participants after the nature and all possible consequences of the study were fully explained to them. Healthy subjects were 18 years of age and older and in good general health. Exclusion criteria were follows: oral inflammatory lesions (including gingivitis and periodontitis), oral cancer diagnosis, soft tissue lesions, and the use of tobacco in the past month. Single cell suspension of MOIL and HOIL were prepared after Collagenase 1A digestion of the mouse tongue/palatal/gingival tissues and human gingival biopsies, respectively.

### Antibodies and Reagents

Purified or fluorochrome conjugated mouse and human α-CD3 (145-2C11), α-CD28, α-CD25 (3C7 and 7D4), CD4, IL-2, IFN-γ, IL-17A, TNF-α, Foxp3, CD45, CD8, CD11C, CD38, HLADR, Phospho-p70 S6 Kinase (Thr389), Phospho-Akt 1 (Ser473), IL-10 (JES5-16E3), IL-6, and p-mTOR antibodies, carboxyfluorescein diacetate succinimidyl ester (CFSE), and Cell Proliferation Dye eFluor 670 (CPD-670) were all purchased from Life Technologies/Thermofisher. PE conjugated F4/80 Monoclonal Antibody (BM8), PerCP-eFluor 710 conjugated Ly-6G Monoclonal Antibody (1A8-Ly6g), APC conjugated CD11b Monoclonal Antibody (M1/70) were all purchased from Ebiosciences/Thermofisher Scientific. Recombinant IL-1β was purchased from BioBasic Inc (Amherst, NY). Human TGF-β1 was purchased from R&D systems. Anti-mouse CD121A (IL1R1) BV421 (1F3F3D4) was purchased from BD Biosciences. Anti-mouse blocking IL-1β blocking antibody was bought from Novus Biologicals. Anti-MyD88-PE antibody was purchased from Santacruz biotechnologies. CD4^+^T cell isolation kit II (Miltenyi Biotec, Auburn) was used for purification of CD4^+^ cells, which were further flow cytometry sorted for naive cells. In some experiments, we used flow cytometry-sorted CD4^+^CD25^+^GFP^+^ T_reg_ cells or CD4^+^CD25^-^ GFP^-^ responder cells from Foxp3^GFP^ reporter mice. The purity of CD44^lo^CD62L^hi^CD25^+^ naive cells was more than 98%. CD4, CD4 naïve cell and T_reg_ magnetic isolation kits were also used and were purchased from Stem cell Technologies (Vancouver, Canada). Mouse cells were cultured in complete RPMI-1640 (Hyclone) supplemented with 10% FCS, 100 U/ml penicillin, 100 µg/ml streptomycin, 2 mM glutamine, 10 mM HEPES, 1 mM sodium pyruvate, and 50 μM β-mercaptoethanol. Some *in vitro* experiments were done using the X-VIVO-15 serum-free media from Lonza/Biowhittaker. Mouse IFN-γ and TNF-α ELISA kits were purchased from Ebiosciences/Thermofisher Scientific. IL-1β and IL-6 ELISA kits were from Boster Bio (Pleasanton, CA). Heat killed *Candida albicans* germ tubes (HKGT) were generated in the laboratory by heat killing the germ tubes at 75°C for 60 min. Germ tubes were prepared by growing blastospores (10*9/ml) in complete RPMI-10 at 37°C with CO_2_ for 4–6 h, or until the budding of germ-tubes.

### Cell Stimulation *In Vitro*

Cells from SPLN, CLN, and MOIL were stimulated in U-bottom 96 well plates using 1 µg/ml of plate-bound α-CD3 and 2 µg/ml α-CD28 antibodies with IL-1β (1–10 ng/ml), TGF-β1 (2 ng/ml), and HKGT for 3–6 days, as indicated. CD90^+^ T cell depleted splenocytes were added as antigen presenting cells (APC), at a T cell: APC ratio of 3:1 during the initiation of cultures, when indicated. In some experiments, CD4^+^ T cells were pre-labeled with CPD-670 before adding in cultures to assess their proliferation. For co-culture T_reg_ suppression assay, CPD670 labeled CD4^+^CD44^lo^CD62L^hi^CD25^+^ naive responder T (T_resp_) cells (3 x 10^4^) were stimulated in U-bottom 96-well plates with 3 x 10^4^ control CD4^+^CD25^-^ cells or 3 x 10^4^ T_reg_ cells using soluble 1 μg/ml α-CD3 and 2 μg/ml α-CD28 antibodies (26).

### Quantitative-Reverse Transcriptase PCR (q-RT PCR)

Naïve CD4^+^ T cells were stimulated as above with soluble 1 μg/ml α-CD3 and 2 μg/ml α-CD28, TGF-β1, HKGT with or without IL-1β for 3 days and were used for q-PCR analyses of ROR-γt, Foxp3, IL-17A, and IFN-γ mRNA. RNA was isolated using an RNA isolation Kit (BioBasic). Removal of genomic DNA from purified RNA was done by DNase (Ambion). Mu-MLV reverse transcriptase, oligo-dT primers (BioBasic), and SYBR Green PCR Kit (BioBasic) and real time PCR machine (Applied Biosystems) were used for cDNA synthesis and qPCR. All primers for PCR (BioBasic) were designed to amplify a coding region within a single exon. The relative amount of mRNA of interest was estimated from its Ct values, which were normalized to the β-actin mRNA levels, assigning values of “1” to unstimulated or “day 0” CD4^+^ T cells that were used as control samples.

### Immunohistochemistry of Proteins and Intracellular Staining of Cytokines

For immunocytochemical periodic acid schiffs (PAS), hematoxylin and eosin (H&E), and Foxp3 antibody histological staining, tongue tissues were cleaned and rinsed with PBS, fixed with 10% formalin overnight, and rehydrated in 70% ethanol overnight. This was followed by sectioning and staining of paraffin sections by the commercial facility (Histoserv, Inc, MD). For single-cell flow cytometry staining, cells were cultured as above and washed in PBS or PBS/BSA before surface staining using the antibodies. For Foxp3 staining, the cells were fixed with Foxp3 fix-perm set (eBioSciences/Thermofisher) after surface staining. Live-Dead viability staining was used to remove dead cells in the analyses. Appropriate un-stain, isotype, secondary antibody, single stain and FMO controls were used. Before intracellular cytokine staining, cultures were re-stimulated with PMA (50 ng/ml) and Ionomycin (500 ng/ml) for 4 h, with brefeldin-A (10 µg/ml) added in last 2 h. For p-IRAK, p-Akt, p-mTOR, and p-70-S6K staining, the cells were washed, fixed and were stained with Phosflow staining kit from BD Biosciences using manufacturer’s protocol.

### Flow Cytometry and Confocal Microscopy

Data was acquired using BD Fortessa cytometers and were analyzed using FlowJo 9.8 or 10.5.3 softwares. Cells were cytospun on the slides, fixed, permeabilized for intracellular flow cytometry and confocal staining.

### Oral *Candida* Infection

Mice were infected as previously described (27, 28). Briefly, they were sublingually infected with tongue abrasion and under anesthesia by placing a 3 mm diameter cotton ball saturated with 1 x 10^7^ *Candida albicans* (SC5314) blastospores for 90 min. Mice were re-infected on day 14 or 15 after primary infection for assessing the secondary immune responses *in vivo.* Mouse body weight was monitored every day until sacrifice. Tongue inflammation scores were assessed as follows: 0 = No fungus and immune infiltrates; 1 = Sparse immune infiltrates; 2 = Sparse fungus with low immune infiltrates; 3 = Frequent fungal hyphae with moderate immune infiltrates; 4 = high immune infiltrates with prominent fungal hyphae; 5 = extensive branched filamentous fungal hyphae, immune infiltrates with epithelial damage (21). Fungal burden (CFU/gm of tongue was assessed by incubating the tongue lysates on sabouraud dextrose agar plates for 24 h (28).

### Statistical Analyses

P values were calculated by Mann-Whitney test in Prism 6.1 (GraphPad Software, Inc.) assuming random distribution. One and Two way ANOVA analyses were also used for grouped analyses. For correlation, spearman analyses were used. P < 0.05* was considered significant.

## Results

### Loss of MyD88 in Foxp3^+^ Cells Reduces T_reg_ Accumulation in Oral Mucosa *In Vivo*

**Figure 1:**
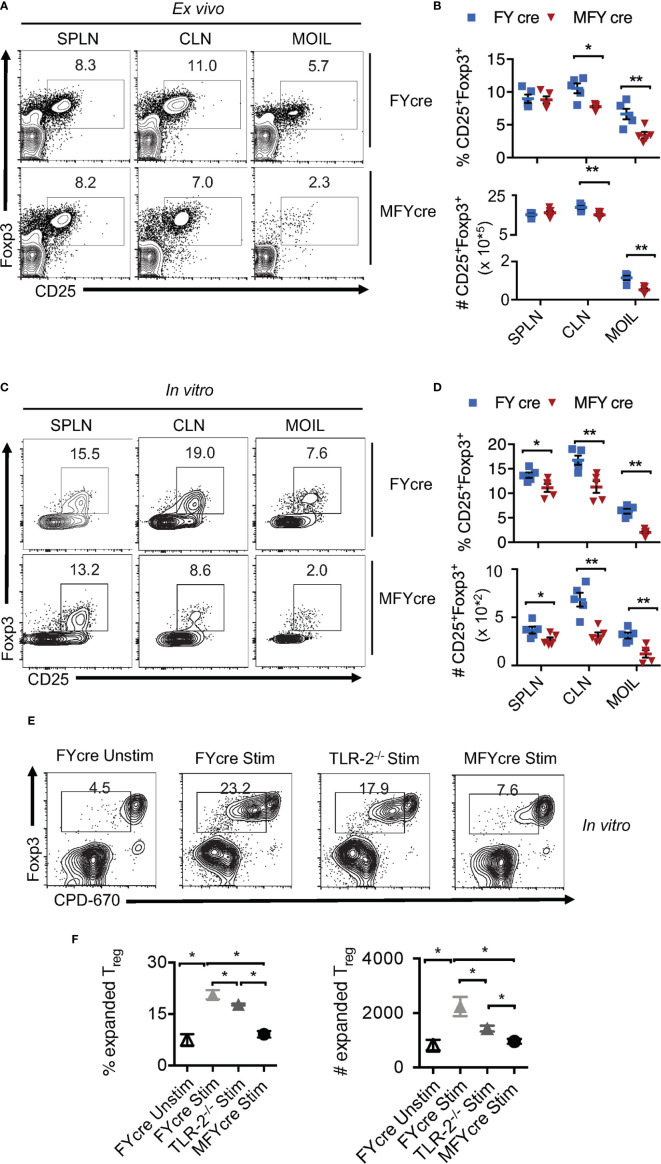
MyD88 deficiency in Foxp3^+^ cells reduces oral mucosa T_reg_ accumulation *in vivo* and during heat killed Candida albicans germ tube (HKGT) activation *in vitro.* Cells were isolated from spleen (SPLN) and cervical lymph nodes (CLN) and mouse oral intra-epithelial lamina propria leukocytes (MOIL) derived from FYcre and MFYcre mice. **(A)** Flow cytometric plots of CD25 and Foxp3 *ex vivo*. **(B)** Statistics of T_reg_ frequency (above) and numbers (below) from individual mouse from FYcre and MFYcre groups *ex vivo*. **(C)** 3 x 10^5^ cells from the indicated tissue were stimulated with α-CD3(1μg/ml, α-CD28 (2μg/ml), TGF-β1 (5 ng/ml) and heat killed *Candida albicans (*CA*)* germ tube (HKGT) (10^7^/ml) for 5 days before assessing CD25 and Foxp3 by flow cytometry. **(D)** Statistics of CD25^+^Foxp3^+^ cell frequency (above) and numbers (below) in cultures stimulated as in **(C)**, from indicated groups (Each data point corresponds to an individual mouse). **(E, F)** MyD88 signaling expands T_regs_. CLN CD4^+^CD25^+^Foxp3-YFP^+^ T_regs_ from FYcre and MFYcre mice, and CD4^+^CD25^+^ T_regs_ from TLR-2^-/-^ were FACS sorted and labelled with CPD-670. 5 x 10^4^ T_regs_ were stimulated with APC as in **C**. Flow cytometric plots showing CPD-670 dilution and Foxp3 **(E)**, and statistics showing T_reg_ expansion **(F)** are depicted. Mean values ± SEM are plotted. (*P < 0.05; Mann Whitney test). Data represent at least triplicate experiments. **P < 0.005.

Although we and others have previously shown that TLR-2/MyD88 signaling can influence mucosal Foxp3^+^ cells (2, 22, 29, 30), T_reg_ specific role of MyD88 was not evaluated during an infection. To this end, we bred *MyD88^fl/fl^* mice with *Foxp3-YFP^cre^* (FYcre) mice and generated MFYcre line in which MyD88 was deleted in CD4^+^Foxp3^+^ cells (**Figures S1A–C**). Although there was a moderate increase in the frequency of CD44^high^ cells in MFYcre mice, they developed normally without any overt oral inflammation in steady-state conditions (**Figure S2**). We examined the proportions of T_regs_ in spleen (SPLN), oral mucosa draining cervical lymph nodes (CLN), and the mouse oral intra-epithelial lamina propria leukocytes (MOIL) derived from tongue and gingival tissues in these mice. We found that the frequency and absolute numbers of CD25^+^Foxp3^+^T_regs_ were significantly lower in CLN and MOIL of MFYcre than in control mice (**Figures 1A, B**). However, these were comparable in cells derived from SPLN. Notably, irrespective of the markers used, different T_reg_ subpopulations, namely, Helios^+^, Helios^-^, Nrp1^+^, and ROR-γt^+^, were all proportionally reduced (**Figure S3A**). A substantial proportion of T_regs_ was ROR-γt^+^ and Helios^-^ in MOIL, which appeared to be diminished in MFYcre mice (**Figure S3B**). Similar to colonic T_regs_ (13), ROR-γt^+^ Helios^-^ Foxp3^+^ cells are likely maintained in a microbiome dependent manner in oral mucosa (27, 31). Based on the previous findings on the proliferative effect of *Candida* on T_regs_ (2, 27, 28), we hypothesized that *Candida* may induce local expansion of mucosal T_regs_, and this expansion might be impaired in MFYcre mice. To test this hypothesis, we treated the cells with heat killed *Candida albicans* germ tube (HKGT) (10^7^/ml), α-CD3 (1 μg/ml, α-CD28 (2 μg/ml), and TGF-β1 (5 ng/ml), to examine the frequency of Foxp3^+^ cells after 5 days. We employed this *in vitro* cell culture system because: 1) We have previously found that HKGT can cause *in vitro* proliferation of T_regs_ in TLR-2 dependent manner; 2) TGF-β1 is important for survival of Foxp3^+^T_regs_ during oral CA infection as well resistance to *Candida in vivo* (2, 32, 33); and 3) Activating T cells in a whole tissue culture system including the local antigen presenting cells (APC) is more physiological than using purified T cell cultures because APC secrete appropriate cytokines in the milieu (2, 32). As expected, HKGT stimulation increased the proportion of Foxp3^+^ cells among CD4^+^ T cells in FYcre cultures compared to those found *ex vivo* (**Figures 1A, C**, upper panel). However, CD25^+^Foxp3^+^ cells from MFYcre mice expanded much less than FYcre T_regs_ (**Figures 1C, D**). These results show that intrinsic MyD88 signaling in Foxp3^+^T_regs_ is a pre-requisite for *Candida* mediated proliferation in TCR activated oral mucosal T_regs_. To determine the effect of APC and TLR-2 signaling, we sorted CD4^+^CD25^+^YFP^+^ cells from FYcre and MFYcre mice and CD4^+^CD25^+^ T_regs_ (> 90% Foxp3^+^) from TLR-2^-/-^ mice. We then stimulated them with C57BL/6 wild-type (WT) APC. We labelled T_regs_ with cell proliferation dye-670 (CPD670) and compared the proliferation of FYcre, TLR-2^-/-^ and MFYcre T_regs_. As expected, FYcre T_regs_ proliferated (**Figure 1E**, top 2 panels), but TLR-2^-/-^ showed a moderate reduction in T_reg_ proliferation (**Figures 1E**, 3^rd^ panel). However, MFYcre T_regs_ were significantly more defective in proliferation (**Figures 1E, F**; compare 3^rd^ row with the last row in **Figure 1E**). Therefore, we rationalized that another MyD88 dependent component that is TLR-2 independent should also induce T_reg_ proliferation. Since IL-1R family members signal through MyD88 (34), MFYcre T_regs_ must lack the ability to signal through cytokines such as IL-1β and IL-33 produced by APC and other cells in the milieu. These cytokines have been previously shown to impact mucosal and tissue T_regs_ (35, 36). Therefore, we tested the effects of IL-1β and IL-33 on T_reg_ proliferation. While IL-1β was able to enhance the proliferation of T_regs_ stimulated as above, IL-33 did not (**Figure S4**, top 4 panels). Thus, these results identified a role for intrinsic MyD88/IL-1β signaling in expanding T_regs_ in conjunction with TCR and TLR-2 activation, which could contribute to the compartmentalized regulation of oral mucosal T_regs_.

### Loss of MyD88 in Foxp3^+^ Cells Exacerbates Oral Inflammation During OPC *In Vivo*

**Figure 2:**
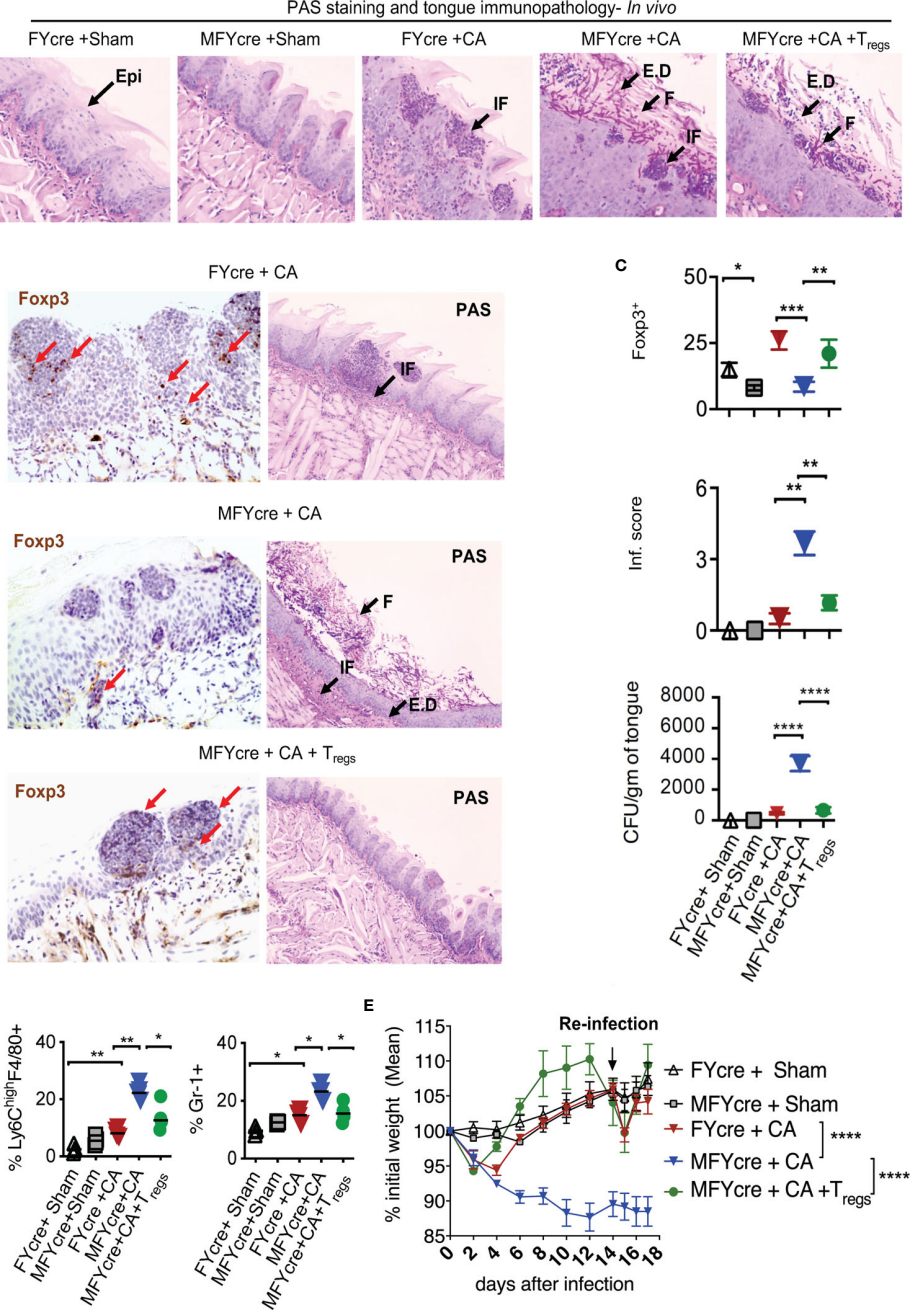
T_reg_ specific deletion of MyD88 reduces results in impaired resistance to oropharyngeal candidiasis (OPC) in mice, and T_reg_ reconstitution reduces fungal burden and immunopathology. FYcre and MFYcre mice were sublingually infected with CA or PBS (Sham) *in vivo* (n= 4-5/group). (MFYcre +CA + T_regs_) group received 1 x 10^6^ CD4^+^CD25^+^GFP^+^ T_regs_ from Foxp3-GFP reporter mice. **(A)** Periodic Acid Schiff’s (PAS) staining was done on tongue sections isolated from mice on day 7 after infection. **(B)** Foxp3 (left) and PAS (right) immunohistochemistry evaluation was performed on tongues derived from mice on day 7 and 18 after infection, respectively. Microscopic images of the slides viewed at 20X magnification (Epi, epithelium; E.D, epithelial damage; F, fungus; IF, immune cell infiltration; Red arrows indicate some of the Foxp3^+^cells). **(C)** Statistical analyses of # Foxp3^+^ (top) cells, inflammation score (middle) from 20X images of the tongues, and fungal burden (CFU/gm of tongue) (bottom panel) assessed in tongue lysates from mice on day 6 or 7 after infection (* P<0.05; Mann Whitney test). **(D)** MOILs were isolated on day-6 after infection and processed for flow cytometric staining of F4/80 and Ly6C (left, **Figure S5B**) and Gr-1 (right, **Figure S5C**). Data represent two experiments. **(E)** Mouse body weight was monitored every other day after infection until the day of sacrifice. The percent weight change in mice in all groups. Mean values ± SEM are plotted. (2 way ANOVA, multiple comparison; alpha= 0.05* significant). At least 3-5 independent experiments showed similar results. **P < 0.005, ***P< 0.0005, ****P < 0.00005.

We next determined if MyD88 signaling in T_regs_ alters the physiological outcome of an oral infection and inflammation. As we have previously shown that T_regs_ are crucial for enhancing anti-fungal Th17 cell response and inflammation control at early and later infection phases respectively (27, 28), we hypothesized that MyD88 deficiency in T_regs_ may increase susceptibility to infection and worsen inflammation during OPC. To validate this hypothesis, we sublingually infected MFYcre mice and control mice with CA *in vivo*. On day 14 post infection, we re-infected them to analyze adaptive immune responses. Control mice were infected with PBS control (sham). As a positive control, in one group of MFYcre mice, we intraperitoneally injected 1 x 10^6^ CD4^+^CD25^+^GFP^+^ T_regs_ from wild-type (WT) Foxp3-GFP reporter mice or CD4^+^CD25^+^ T_regs_ from congenic CD45.1 mice 2 weeks prior to the infection. Seven days after the infection, we assessed the fungal burden in the tongue using Periodic Acid Schiff’s (PAS) histochemical staining, which detects fungal hyphae in tongue sections. As anticipated, sham infected control mice did not show fungal presence (**Figure 2A**, **S5A**, 1^st^ and 2^nd^ panels). MFYcre mice showed substantially more hyphae persisting in the tongue compared to FYcre control mice in infected groups (**Figure 2A**, **S5A**, 3^rd^ and 4^th^ panels). MFYcre mice that received adoptively transferred wildtype T_regs_, however, had fewer hyphae compared to untreated MFYcre mice (**Figure 2A**, **S5A**, 5^th^ panel). Immunohistochemistry showed increased inflammatory infiltrates and reduced numbers of T_regs_ correlating in tongues of MFYcre mice compared to control FYcre mice (**Figure 2B**, left and right, top 2 panels). Determining the fungal growth in tongue lysates also confirmed that reduction of Foxp3^+^ T_regs_ correlated with increased fungal burden in MFYcre mice (**Figure 2C**, 3^rd^ panel). T_reg_ injected MFYcre mice showed increased infiltrating T_regs_ and concomitant decrease in fungal burden (**Figure 2B**, left and right, bottom panel, **Figure 2C**, three panels). Heightened persistence of inflammatory F4/80^+^Ly6-C^high^ macrophages and Gr-1^+^ neutrophils in oral mucosa, even on day 7 after infection, demonstrated tongue immunopathology in infected MFYcre mice (**Figure 2D**, left and right, **S5B, C**). These mice also showed worse weight loss following primary infection and re-infection (**Figure 2E**). Thus, we inferred that the absence of MyD88 in T_regs_ led to an increased fungal burden and continued tissue inflammation, which may trigger a positive feedback loop leading to persistent infection burden.

### T_reg_ Specific Deletion of MyD88 Diminishes IL-17A but Increases IFN-γ Expression in Effector Cells and Foxp3^+^ Cells During Infection

**Figure 3:**
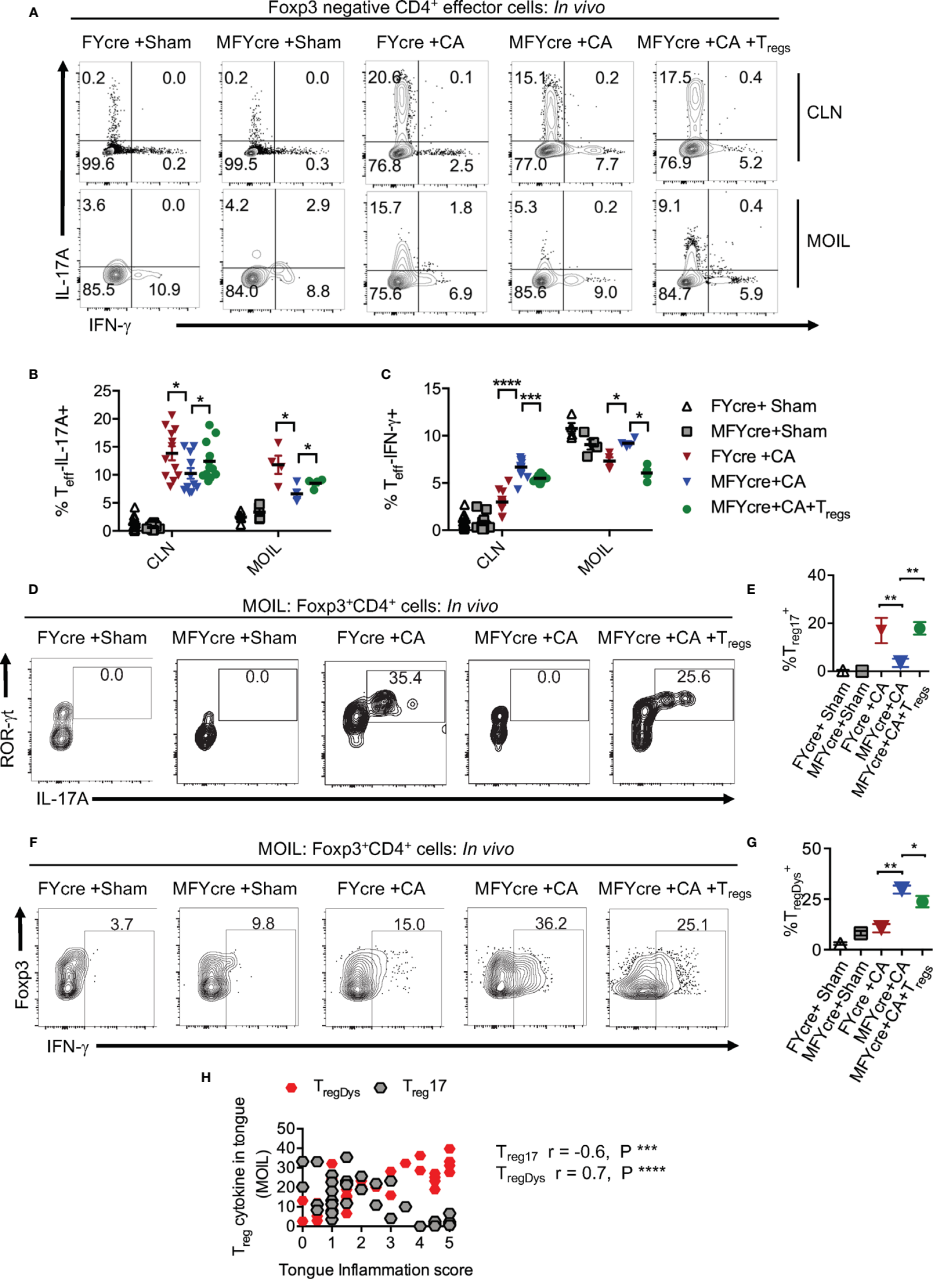
Loss of MyD88 in T_regs_ diminishes IL-17A and increases IFN- γ expression in effector cells and Foxp3^+^ cells *in vivo* during oropharyngeal candidiasis (OPC) infection. FYcre and MFYcre mice were infected with sham control or CA as in **Figure 2** (n= 4–6/group). On day 3 after infection, cells from cervical lymph nodes (CLN) and mouse oral intra-epithelial and lamina propria leukocytes (MOIL) were restimulated with PMA-ionomycin to assess intracellular proteins. MOIL cells pooled from 2 mice were used for flow cytometric analyses. **(A)** Flow plots of IL-17A and IFN-γ, gated on CD3^+^CD4^+^ Foxp3^-^ effector (T_eff_) cells. Statistical analyses of T_eff_ IL-17A^+^ **(B)**, and T_eff_ IFN-γ^+^ **(C)**. Flow plots of ROR-γt and IL-17A **(D)**, Foxp3 and IFN-γ, **(F)** gated on CD3^+^CD4^+^ Foxp3^+^T_reg_ cells, and statistical analyses of the proportion of ROR-γt^+^IL-17A^+^ T_regs_ (% T_reg17_) **(E)**, and T_reg_ IFN-γ^+^ (% T_regDys_) **(G)**, in different groups are shown. For statistical analyses, data are pooled from two experiments. Each data point represents one mouse in CLN and 2 mice in MOIL. Mean values ± SEM are plotted. **(H)** Correlation curve was plotted using values from tongue inflammation score, %MOIL T_reg_17 and %MOIL T_regDys_ in infected mouse groups. These data represent three independent experiments showing similar results. *P < 0.05, ***P < 0.005, ***P < 0.0005, ****P < 0.00005.

**Figure 4:**
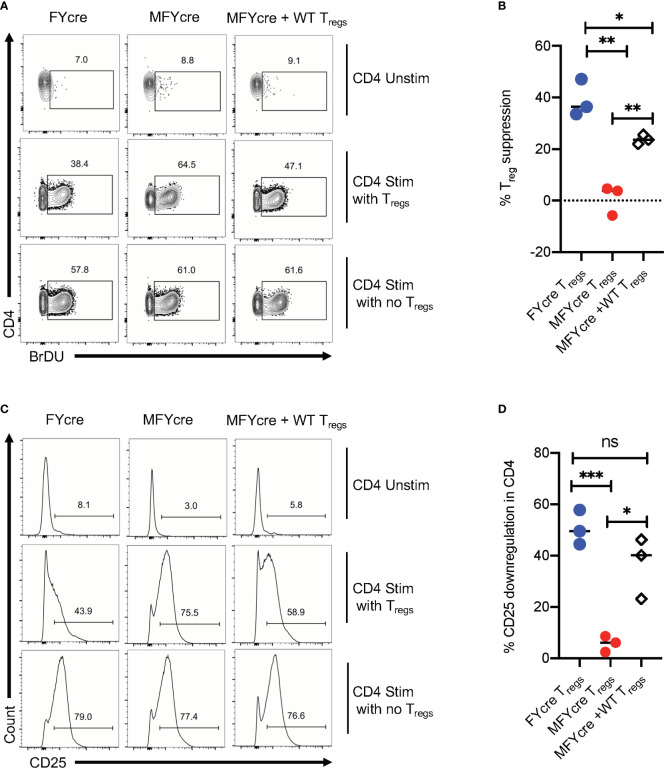
CD25^+^Foxp3^+^ cells lacking MyD88 in infected mice lack suppressive functions *in vitro*. FYcre and MFYcre mice were infected with sham control or CA as in **Figure 2** (n= 4–6/group). On day 6 after infection, cells from cervical lymph nodes (CLN) and mouse oral intra-epithelial and lamina propria leukocytes (MOIL) of FYcre + CA, MFYcre + CA and MFYcre + CA +T_regs_ groups were collected for *in vitro* stimulation with α-CD3 and α-CD28 antibodies (CD4 Stim with T_regs_) for 3–4 days. Some cells each mouse group were stimulated after the removal of CD4^+^CD25^+^ T_regs_ (CD4 Stim with no T_regs_). A control culture was plated with no restimulation (CD4 Unstim). BrDU was added in the last 24 h before harvesting the cells for fixation and flow cytometry for BrDU **(A, B)** and CD25 **(C, D)**. Flow cytometry gating excluded Foxp3^+^ T_reg_ cells in cultures. T_reg_ suppression was measured by % BrDU+ proliferating cells as normalized to “CD4 Stim with no T_regs_” cultures. Mean values ± SEM from 3 independent experiments are plotted. * P< 0.05; 2 way ANOVA and Unpaired 2-tailed students “t” tests. **P < 0.005, ***P < 0.0005. NS, non-significant.

To determine how oral mucosal T_regs_ contribute to immune cell changes during infection, we analyzed the CD4^+^T cell response in CLN and MOIL in MFYcre mice infected with CA. Examining IL-17A and IFN-γ in Foxp3-negative effector CD4^+^ T cells on day 3 after re-infection, we found that both sham groups had negligible but comparable levels of cytokine producers. CA infected control mice specifically expressed IL-17A (y-axis) and very little IFN-γ (x- axis) in response to the infection (**Figures 3A-C**). Effector T cells in MFYcre mice, however, produced slightly reduced IL-17A but increased IFN-γ, suggesting a Th1 skewed response when compared to FYcre mice (**Figures 3A, C**). These changes were not observed in SPLN, indicating that immune cell changes were in response to local infection in oral mucosa, as shown previously (27). High proportions of Th17 cells and IFN-γ expressing effector cells (Th*) were observed in MFYcre mice even 26 days after primary infection, indicating a persistent inflammation in these mice. Although there were no differences in IL-10 expression in CD4^+^Foxp3^+^ T cell compartment between these groups of mice (**Figure S6A**), MyD88 deficiency in T_regs_ correlates with tongue pathology (**Figure S6B**, **Figure 2**). MFYcre mice that received WT T_reg_ (from congenic CD45.1) injection had significantly lower IFN-γ producing effector cells at all time-points after infection (**Figures 3A, C**, **S6B**, last panel). These results showed that T_reg_-intrinsic MyD88 signaling is required not only for its proliferation, but also for its ability to control inflammatory IFN-γ producing effector cells. Presence of T_reg_17 cells is known to correlate with the control of immunopathology during OPC (31). While short-chain fatty acids and TLR-2 ligands were involved in promoting T_reg_17 cells through independent mechanisms, T_reg_-intrinsic MyD88 signaling was not explored (2, 27, 37). Therefore, we examined the proportions of T_reg_17 cells in the oral mucosa, and found that Foxp3^+^ cells deficient in MyD88 signaling did not show ROR-γt and IL-17A expression during infection (**Figure 3D**). However, CD45.1 WT T_regs_ in MFYcre mice showed ROR-γt and IL-17A expression (**Figures 3D, E**). The proportion of IFN-γ expressing Foxp3^+^ cells was significantly higher in MFYcre mice than in control mice (**Figures 3F**, 1–4 panels, **G**). Clearly, T_reg_ intrinsic MyD88 signaling was essential for restraining IFN-γ expression in Foxp3^+^ cells because CD45.1^+^Foxp3^+^ cells in MFYcre mice reconstituted with WT T_regs_ expressed lower levels of IFN-γ (**Figures 3F, G**). Previously, IFN-γ expression in T_regs_ has been shown to be associated with human inflammatory diseases and dysfunction in T_reg_ cells (38). Our results are also consistent with this notion, because T_reg_IFN-γ appeared to be dysfunctional (T_regDys_) and positively correlated with increased immunopathology in infected mice (**Figures 2**, **3H**). In contrast, increased T_reg_17 cells positively correlated with lower inflammation score in infected mice (**Figure 3H**). These results show that T_reg_-intrinsic MyD88 signaling is critical in controlling their functions by differentially modulating their expression of IL-17A and IFN-γ. Defects in this signaling converts host defense Th17 response in to an immunopathological response during oral *Candida* mucosal infection.

### Loss of MyD88 in T_regs_ Abrogates Their Suppressive Activity *In Vitro*

The above-mentioned results suggest that the absence of MyD88 in Foxp3^+^T_regs_ not only impair early Th17 responses, but also render T_regs_ unable to control excessive CD4^+^T cell responses during the resolution phase of infection. To further verify this possibility, we determined the ability of FYcre and MFYcre T_regs_ isolated from infected mice to suppress CD4^+^T cells *in vitro*. To this end, we isolated CLN and MOIL from infected mice at a late phase of infection and re-stimulated them in the presence or absence of T_regs_ in cultures. For removal of T_regs_ in cultures, we depleted CD4^+^CD25^+^ cells before re-stimulation with α-CD3 and α-CD28 antibodies. T_reg_ depletion led to a decrease of CD25^+^Foxp3^+^ cells from 24.8% to 2.1%, 9.2% to 1.4%, and 18.6% to 2.3% in FYcre, MFYcre, and MFYcre + WT T_reg_ cultures respectively (data not shown). We then examined the proliferation of Foxp3 negative effector CD4^+^ T cells by 5-bromo-2’-deoxyuridine (BrdU) labeling assay. The cells with no re-stimulation did not undergo proliferation (Unstim, **Figures 4A**, top row, B). As expected, depletion of FYcre T_regs,_ but not MFYcre T_regs_ *in vitro*, significantly increased the proliferation of responding CD4^+^ T cells in their respective cultures (**Figures 4A**, 1^st^ 2 columns, **B**). More importantly, WT T_regs_ that were injected *in vivo* also retained their suppressive capacity *in vitro* and inhibited the proliferation of MFYcre CD4^+^ T cells (**Figures 4A**, 3^rd^ column, **B**). We and others have previously shown that T_regs_ can downmodulate the sensitivity of effector cells to IL-2 by reducing their IL-2 receptor (CD25) expression (26, 28, 39). Therefore, we determined the expression of CD25 on Foxp3^negative^ effector cells in these cultures. As expected T_regs_ downmodulated CD25 expression in FYcre cultures and MFYcre cultures with WT T_regs_ (**Figures 4C**, 1^st^ and 3^rd^ columns, **D**). However, depletion of MFYcre T_regs_ did not affect CD25 expression in effector cells (**Figures 4C**, 2^nd^ column, **D**), suggesting that MFYcre T_regs_ had impaired ability to control CD25 expression and excessive CD4 T cell responses. Collectively, these data confirm a nonredundant role of MyD88 in immunomodulatory function of Foxp3^+^ cells during the later phase of the infection *in vivo.*

### Induction and Proliferation of T_reg_17 and T_regDys_ Cells Are Driven by IL-1β and IL-6, Respectively

**Figure 5:**
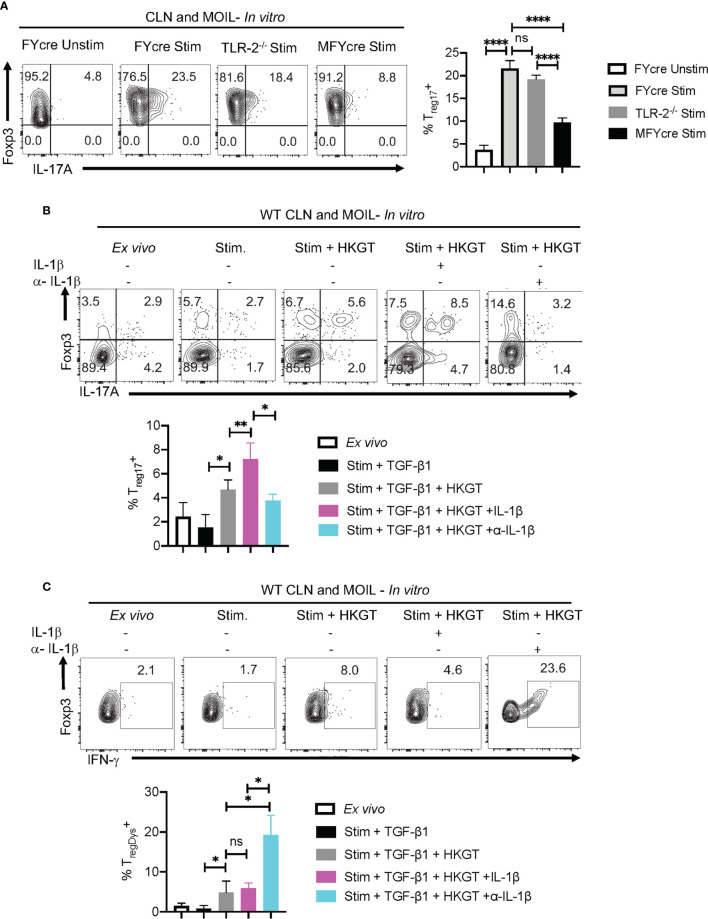
IL-1β promotes T_reg_17 cells and constrains induction of T_regDys_ cells in *Candida* activated oral mucosal cells *in vitro*. **(A)** HKGT mediated T_reg_17 induction is slightly reduced in TLR-2^-/-^ T_regs_, but significantly lower in MFYcre T_regs_ *in vitro*. Pooled cervical lymph nodes (CLN) and mouse oral intra-epithelial and lamina propria leukocytes (MOIL) cells from FYcre and MFYcre were stimulated with heat killed *Candida albicans* germ tube (HKGT) as in **Figure 1C** for 5 days and were restimulated with PMA-Ionomycin for flow cytometry assessment. For TLR-2^-/-^ cultures, T_regs_ were purified from TLR-2^-/-^ mice and stimulated with WT APC from T cell depleted CLN and MOIL cells. Flow cytometry plots showing Foxp3 and IL-17A expression (gated on CD3^+^CD4^+^Foxp3^+^ cells). **(B, C)** WT CLN and MOIL cells were examined *ex vivo* or stimulated with HKGT as in **Figure 1C**, in the presence of IL-1β (10 ng/ml) or α- IL-β antibody (10 μg/ml) for 5 days. Flow cytometry plots showing Foxp3 and IL-17A expression (gated on CD3^+^CD4^+^ cells) (**B**, top) and statistical data points from experimental replicates (**B**, bottom). Flow plots showing Foxp3 and IFN-γ expression (gated on CD3^+^CD4^+^Foxp3^+^ cells) **(C)** and statistical data points from experimental replicates (**C**, bottom). NS, non-significant. *P < 0.005, **P < 0.0005, ****P < 0.00005.

**Figure 6:**
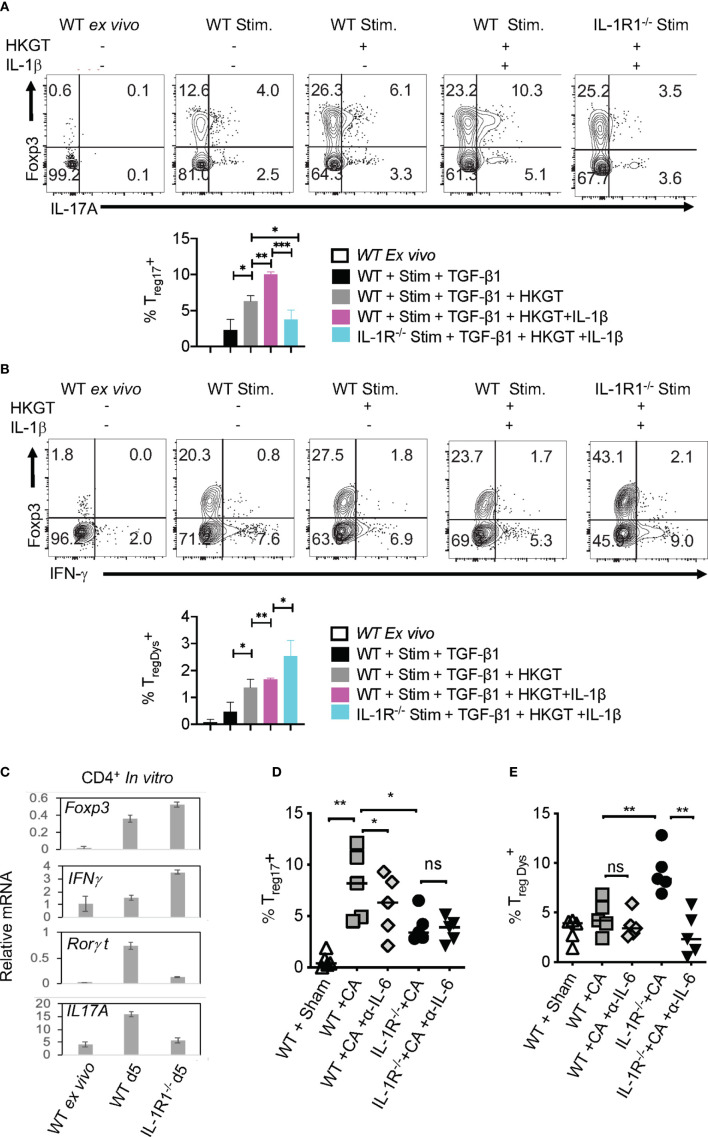
Loss of endogenous IL-1R signaling diminishes T_reg_17 but increases T_regDys_ cells in CD4^+^ T cells *in vitro*. IL-6 expands T_regDys_ cells in the absence of IL-1β. Naïve CD4^+^ T cells from WT C57BL/6 and IL-1R (IL-1R1^-/-^) knockout mice were stimulated with heat killed *Candida albicans* germ tube (HKGT) and TGF-β1 for 5 days as in **Figure 1C** with WT APC and stained for Foxp3, IL-17A (**A**, top and bottom), and Foxp3 and IFN-γ (**B**, top and bottom) (gated on CD3^+^CD4^+^ cells). **(C)** Naïve CD4^+^ T cells from WT C57BL/6 and IL-1R (IL-1R1^-/-^) knockout mice were stimulated with HKGT and TGF-β1 for 3 days. CD4 T cells were purified from these cultures for qPCR assessment of indicated transcripts. **(D, E)** WT and IL-1R1^-/-^ mice were orally infected with CA, and cervical lymph nodes (CLN) and mouse oral intra-epithelial and lamina propria leukocytes (MOIL) were collected 3 days after infection (n=5/group) for HKGT restimulation for 2 days and flow cytometry. Statistical analyses of Foxp3^+^ROR-γt^+^IL-17A^+^ **(D)**, and Foxp3^+^ IFN-γ^+^ **(E)**, expressing cells after PMA/Iono restimulation for 4 h *in vitro* (gated on CD3^+^CD4^+^ cells). α- IL-6 antibody (10 μg/ml) was added in some cultures. Data are representative of at least 3 independent experiments. NS, non-significant. *P < 0.05, **P < 0.005, ***P < 0.0005.

Based on our *in vivo* experiments that showed MyD88’s role in promoting T_reg_17 cells and inhibiting T_regDys_ cells, we then examined how MyD88 deficiency affects T_regs_ during *Candida* stimulation *in vitro*. Therefore, we stimulated CLN and MOIL cells from FYcre and MFYcre mice with HKGT as in **Figure 1C**, and determined T_reg_17 induction. As a control, we also stimulated purified TLR-2^-/-^ T_regs_ with HKGT and wildtype APC. Although HKGT mediated T_reg_17 induction was unaffected in TLR-2^-/-^ T_regs_, it was significantly reduced in T_regs_ lacking MyD88 *in vitro* (**Figure 5A**, left and right). This indicated that HKGT mediated T_reg_17 induction is dependent on MyD88 expression and not TLR-2 expression in T_regs_, suggesting the involvement of IL-1/MyD88 signaling. Because we observed that IL-1β signaling is involved in *Candida* dependent T_reg_ proliferation (**Figure S4**) and Th17 cell induction in mucosa (40), we focused on IL-1β, which we propose to be secreted by APC in the milieu. We stimulated CLN/MOIL cells with TCR activating antibodies along with TGF-β1, HKGT, and IL-1β, alone or in combination (**Figure S7**). We analyzed Foxp3 (y-axis) and IL-17A (x-axis) expression in CD4^+^ T cells on day 5 after stimulation. While HKGT increases the frequency of Foxp3^+^ cells and T_reg_17 cells, IL-1β, and TGF-β1 alone did not induce T_reg_17 cells. However, a combination of these reagents synergistically promoted an increase in the frequency of T_reg_17 cells (**Figure S7**). While adding exogenous IL-1β increased the frequency of T_reg_17 cells, blocking IL-1β significantly diminished T_reg_17 cells (**Figure 5B**, top and bottom).This result suggested that APC produce endogenous IL-1β. As expected, IL-1β dependent IL-17A expression was also observed in Foxp3-negative cell compartments (**Figure 5B**). Surprisingly, while exogenous IL-1β decreased IFN-γ expression in Foxp3^+^ cells, antibody mediated blocking of endogenous IL-1β heightened the frequency of IFN-γ expressing Foxp3^+^ cells (**Figure 5C**). To further evaluate the involvement of IL-1 receptor signaling in induction and proliferation of Foxp3^+^T_regs_, we stimulated naïve CD4^+^ T cells from WT C57BL/6 and IL-1R knockout mice (IL-1R1^-/-^) with HKGT along with APC from CLN. HKGT promoted T_reg_17 cells in IL-1β/TGF-β1 stimulated WT cells but not in IL-1R1^-/-^ cells (**Figure 6A**). These results demonstrated the role of endogenous IL-1β in promoting IL-17A expression in induced Foxp3^+^ cells (**Figure 6A**, upper right quadrants, top, and bottom). Although the effects were moderate, the frequency of IFN-γ was consistently higher in IL-1R1^-/-^ CD4^+^ T cells than in WT cells (**Figure 6B**, upper right quadrants, top, and bottom). Corroborating these flow cytometry data, quantitative PCR (qPCR) of IL-1R1^-/-^ CD4^+^ T cells from these cultures showed relatively lower abundance of ROR-γt and IL-17A mRNA, but slightly higher levels of Foxp3 and IFN-γ mRNA than WT CD4^+^ T cells (**Figure 6C**). To validate IL-1β effects *in vivo*, we orally infected WT and IL-1R1^-/-^ mice with CA and examined Foxp3^+^ cells in MOIL three days after infection and *in vitro* restimulation. While T_reg_ proportions were comparable in uninfected mice (**Figure S8**), infected IL-1R1^-/-^ mice displayed strikingly lower frequencies of T_reg_17 cells and higher T_regDys_ cell proportions compared to WT cells *in vivo* (**Figures 6D, E**). Because IL-6 is also required for Th17 cell differentiation, we determined the role of IL-6 in IL-17A expression in T_regs_. Surprisingly, blocking IL-6 using an antibody reduced T_reg_17 proportions only in WT, but not in IL-1R1^-/-^ cultures. However, it significantly diminished the frequency of T_regDys_ in IL-1R1^-/-^ cells (**Figures 6D, E**). Although mechanisms of contrasting effects of IL-6 and IL-1β are unclear at the molecular level, our data show that IL-1β is key in generating T_reg_17 cells and inhibiting IL-6 dependent IFN-γ expression in Foxp3^+^ cells during *Candida* stimulation. Collectively, the data presented herein confirm the expected role of IL-1β and IL-6 signaling in CD4^+^ T cells, but they also reveal new roles for these signaling pathways in regulating T_reg_ functional plasticity.

### IL-1β Induced T_reg17_ Cells Requires m-TOR Activation

**Figure 7:**
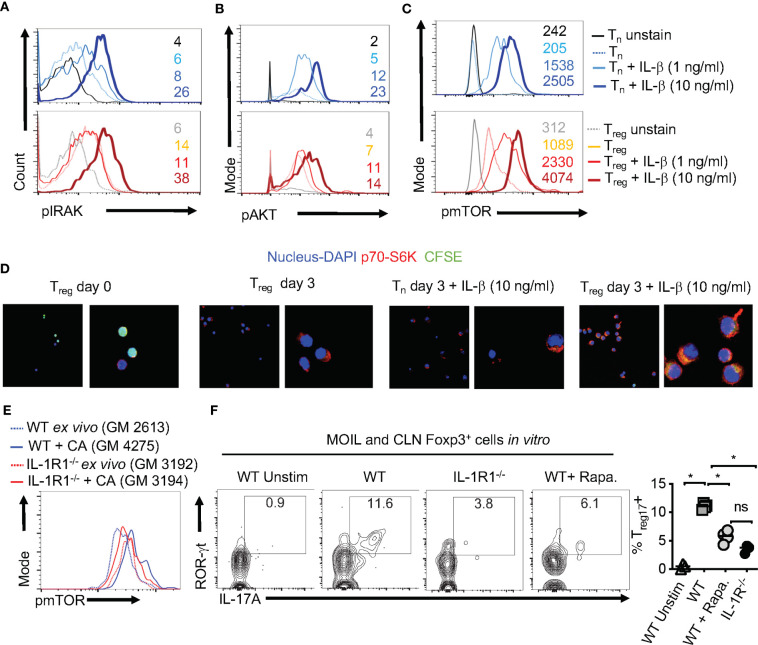
IL-1β induces IRAK, Akt, TOR, and p-70-S6K activation in T_regs_ **(A) **Naïve CD4^+^ T (T_n_) and CD4^+^CD25^+^T_reg_ cells were CFSE labeled and stimulated as in **Figure 1C** for 3 days without or with IL-1β. Cells were restimulated with 10 ng/ml of IL-1β for 4 h before fixation and staining for p-IRAK **(A)**, p-Akt **(B)**, p-mTOR **(C)**, and p-70-S6K **(D)** for flow cytometry analyses, or cytospun on slides for confocal analyses. **(E)** Mouse oral intra-epithelial and lamina propria leukocytes (MOILs) were collected from oral CA infected WT and IL-1R1^-/-^ mice 3 days after infection (n=5/group) and stained for p-mTOR as in **(C)**. (**F**, left and right) Pooled cervical lymph nodes (CLN) and MOIL cells were stimulated with HKGT as in **Figure 1C** for 5 days with or without Rapamycin (1 ng/ml Rapa). Cells were restimulated with PMA/Ionomycin before intracellular staining and flow cytometry analyses (gated on Foxp3^+^ cells). Geometric mean fluorescence intensities (GM) are shown with the histogram plots. Data represent triplicate experiments with similar results. NS, non-significant. *P < 0.05.

Considering the direct effect of IL-1β/MyD88 axis on CD4^+^ T cells in inducing T_reg_17 cells, we next determined the biochemical basis underlying IL-1β signaling in purified naïve cells and T_regs_ *in vitro*. We labeled the cells with carboxyfluorescein diacetate succinimidyl ester (CFSE), added 1-10 ng/ml of IL-1β in some cultures at the beginning of stimulation, and assessed them after 3 days. First, we analyzed IL-1R-associated kinase 4 (IRAK) phosphorylation (p-IRAK4) as a surrogate for MyD88 signaling, and found that T_reg_ cells had slightly higher levels of p-IRAK-4 expression with HKGT alone when compared to naive cells (**Figure 7A**). This result is consistent with an observed TLR-2 expression on T_regs_ (2). The addition of IL-1β further increased p-IRAK4 expression, both in naïve and T_reg_ cells in a concentration dependent manner (**Figure 7A**). Since IL-1β has been previously implicated in NF-κB activation in immune cells (41), we examined whether it impacts activation and nuclear translocation of NF-κB in HKGT stimulated T cells. Confocal microscopic analyses revealed that IL-1β did not promote NF-κB translocation in activated naïve and T_reg_ cells (**Figure S9**). Because IL-1β can affect Akt/mTOR metabolic signaling proteins in T cells (42, 43), we turned to examining phosphorylated-Akt (p-Akt) and phosphorylated-mammalian target of Rapamycin (p-mTOR), which are the activated forms of these proteins. IL-1β was able to increase p-Akt and p-mTOR expression in naïve and Foxp3^+^ CD4^+^ T cells in a concentration dependent manner (**Figures 7B, C**). We then determined the levels of a target protein of mTOR, namely phosphorylated-70-S6K (p-70-S6K), by confocal analyses of CFSE labeled T_regs_. While TCR and HKGT stimulation alone caused CFSE dilution (**Figure 7D**, first two panels), IL-1β further enhanced p-70-S6K in proliferating Foxp3^+^ cells in a concentration dependent manner (**Figure 7D**, last two panels). To evaluate these IL-1β mediated effects *in vivo*, we analyzed the expression of mTOR in Foxp3^+^ cells in oral mucosa (MOIL) derived *ex vivo* and from *Candida* infected WT and IL-1R1^-/-^ mice. *Candida* infection promoted mTOR phospohorylation in WT but not in IL-1R1^-/-^ T_regs_ *in vivo* (**Figure 7E**). Akt/mTOR signaling is well established in T_reg_ and Th17 homeostasis and functions in other settings (31, 44–46). Therefore, we hypothesized that IL-1β signaling might enhance IL-17A in Foxp3^+^ cells in an mTOR dependent manner in the context of *Candida* activation. To this end, we determined IL-17A in HKGT/IL-1β stimulated CLN/MOIL cells in the presence and absence of an mTOR inhibitor Rapamycin (Rapa) *in vitro.* Gating on Foxp3^+^ cells in these cultures revealed that HKGT/IL-1β dependent T_reg_17 induction was blunted in Rapa treated cells (**Figure 7F**, left and right). Collectively, these results showed that IL-1β/IL-1R signaling is required for promoting mTOR activation and IL-17A induction in Foxp3^+^ cells during *Candida* infection in oral mucosa.

### *Candida* Infected Aged Mice Display Immunopathology, Lower IL-1β Induction, but Excessive Secretion of IL-6

**Figure 8:**
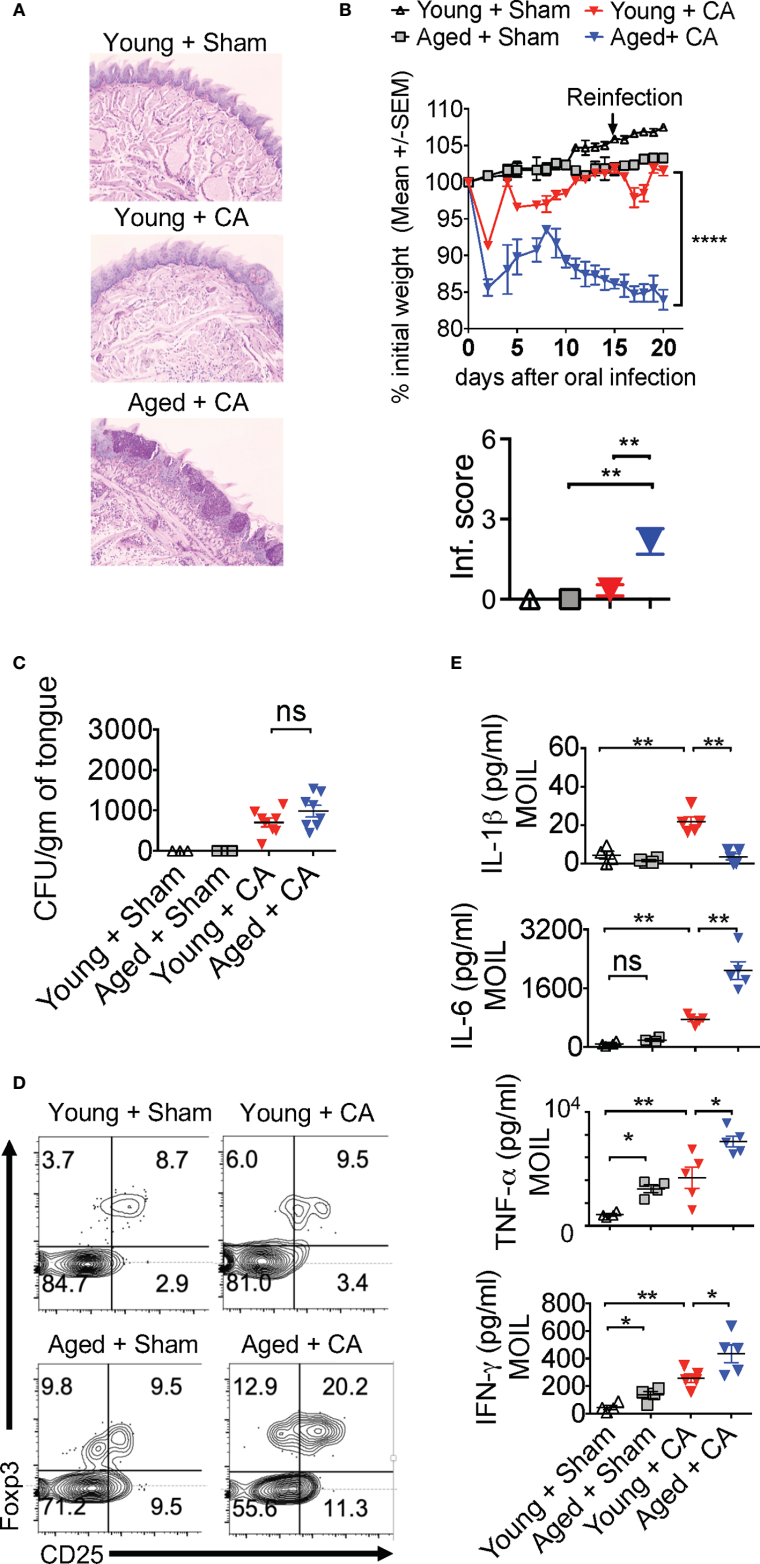
Aged mice show diminished IL-1β but excessive IL-6 expression and immunopathology during OPC. Young (6 weeks) and Aged (age 12–18 months) mice were sublingually infected and re-infected with CA or Sham (n= 4–5/group) as in **Figures 2** and **3**. Histological analyses of PAS staining **(A)**, body weight (**B** above), tongue inflammation score (**B**, below) and fungal burden in tongue lysates **(C)** on day 7 of infection are shown. **(D)** Flow cytometric analyses of oral mucosal CD3^+^CD4^+^ gated cells for CD25 and Foxp3; Geometric means of CD25 in Foxp3+ cells: Young + Sham= 776; Young +CA= 851; Aged + Sham= 334; Aged + CA=618. **(E)** Supernatants were collected from MOIL cells restimulated with PMA/Ionomycin, and IL-1β (on day 2 after infection), and IFN-γ, IL-6, and TNF-α levels (day 5 after infection) were quantified by ELISA. NS, non-significant. *P < 0.005, **P < 0.0005, ****P < 0.00005.

Our results imply that *Candida* infection in the context of IL-1β/IL-6 imbalance may lead to an inappropriate accrual of dysfunctional IFN-γ^+^Foxp3^+^ cells. Interestingly along these lines, aging is associated with elevated levels of serum IL-6 and IFN-γ^+^cells, increased prevalence of Foxp3^+^cells in blood, immune system decline (immunosenescence), and an exaggerated inflammatory state (inflammaging) (47–53). Therefore, we next investigated these cytokines in the context of MyD88/IL-1β signaling and T_reg_ functions during *Candida* infection in aged mice. To this end, we sublingually infected young (6-8 weeks of age) and aged (12–18 months of age) mice with *Candida* and monitored their immunopathology, weight loss and fungal burden. Examining the inflammatory infiltrates on day 20 after initial infection showed there was increased immunopathology in infected aged mice when compared to younger counterparts (**Figure 8A**). Infection caused significantly more weight loss (**Figure 8B**, top) and persistence of infiltrating inflammatory cells (Inf. score) in aged mice than in young mice (**Figure 8B**, bottom). Remarkably, PAS histochemical staining and culturing tongue lysates to determine the fungal growth revealed that there was no significant increase in fungal burden in aged tongues (**Figure 8C**). These results indicated that both young and aged mice were capable of clearing the fungal infection in a similar manner (**Figure 8C**). We then analyzed the CD4^+^ T cells in oral mucosal tongue tissues. The basal levels of Foxp3^+^ cells were proportionally higher in SPLN, CLN and MOIL in aged uninfected mice (**Figures 8D**, **S10**). However, we observed blunted expression of CD25 in aged Foxp3^+^ cells (**Figure 8D**, X axis; see legend). Upon OPC infection, we found that both the frequency and number of Foxp3^+^ cells increased in both the groups, but at significantly higher levels in aged mice than in young mice (**Figures 8D**, **S10**). Finally, to evaluate the role of IL-1β and IL-6 imbalance in aged mice, we examined the levels of these cytokines along with other pro-inflammatory cytokines using Enzyme-linked immunosorbent assay (ELISA). ELISA of oral tongue tissues (MOIL) 2 days after infection showed that while infection promoted the expression of IL-1β in MOIL of young mice, cells derived from aged MOIL produced little or no IL-1β (**Figure 8E**, top panel). This result was observed in MOIL and CLN, but not in SPLN, again highlighting a compartmentalized and infection- mediated effects in T cells of oral mucosa (**Figure S11A**). However, concurrent to increased immunopathology in aged mice, aged MOIL supernatants revealed highly elevated levels of pro-inflammatory cytokines such as IL-6, TNF- α, and IFN-γ, substantiating the presence of oral immune hyperactivation in aged mice (**Figure 8E** rows 2–4, **S11B**). Collectively, these results show that tongue immunopathology could be ascribed to an impairment of IL-1β and excessive production of IL-6 during oral *Candida* infection in aged mice.

### Aged Mice Display Dysfunction in Foxp3^+^ Cells During *Candida* Infection

**Figure 9:**
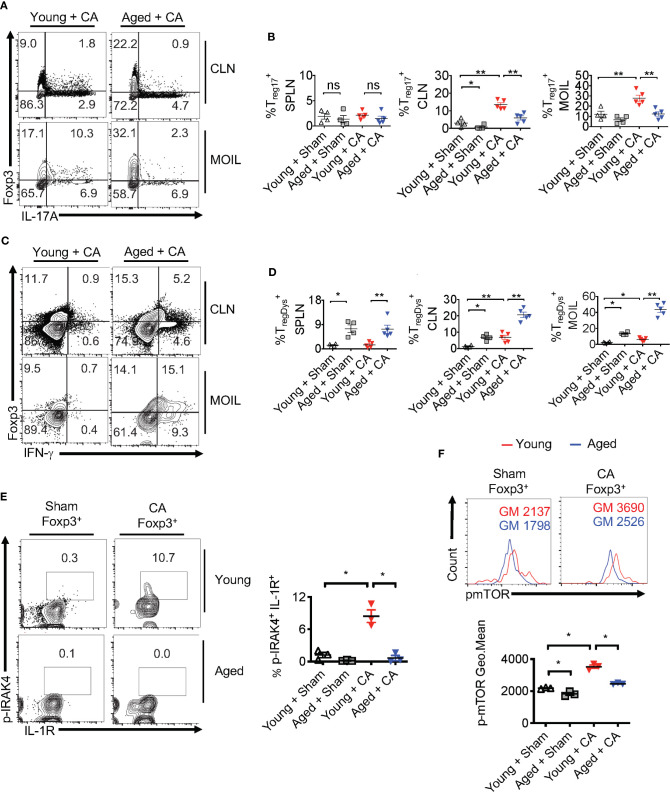
T_reg_17 and T_regDys_ alterations are accompanied by loss of IRAK-4 and mTOR activation in T_regs_ in infected aged mice. Young and Aged mice were infected with CA or Sham as in **Figure 8**. Flow cytometric analyses of CD3^+^CD4^+^ gated cells for IL-17A and Foxp3 **(A, B)**, IFN-γ and Foxp3 **(C, D)**, on day 5 after infection. Statistical analyses of Foxp3^+^ IL-17A^+^ (% T_reg_17) **(B)**, and Foxp3^+^ IFN-γ^+^ (% T_regDys_) **(D)**, using data points from **(A, B)**, respectively. **(E, F)** Mice were infected for 2 days. Flow cytometry analyses of CD3^+^CD4^+^ Foxp3^+^ gated cells for p-IRAK and IL-1R (**E**, left and right) and p-mTOR (**F**, top and bottom) in Foxp3^+^T_regs_. Geometric mean fluorescence intensities (GM) are indicated in the histogram plots. Mean values ± SEM are plotted in statistical analyses. These data show one of four independent experiments showing similar results. (*P < 0.05; Mann Whitney test). **P < 0.005. NS, non-significant.

Despite increased proportions of Foxp3^+^ cells, loss of IL-1β may render aged Foxp3^+^ cells dysfunctional, similar to MyD88^-/-^ T_regs_ (**Figures 2**–**4**). This may provide a positive feedback inflammatory loop to further exacerbate immunopathology during infection. To validate this hypothesis, we analyzed T_reg_ cytokine expression using flow cytometry. Remarkably, the proportion of T_reg_17 cells was significantly lower in CLN and MOIL in aged mice compared to young mice (**Figures 9A**, upper right quadrants, **B**). There were no significant differences between the young and aged groups in IL-17A production by non T_regs_ (Foxp3 negative; Th17) (**Figures 9A**, lower right quadrants, **B**). Foxp3^+^ cells from young mice produced very little IFN-γ, while those derived from aged mice displayed higher levels of IFN-γ production (**Figures 9C**, upper right quadrants, **D**). This effect was much more pronounced in oral mucosa of infected aged mice, reminiscent to MFYcre T_regs_ *in vivo* (**Figure 3**). Both Foxp3^+^ cells and IFN-γ^+^ effector cells were present at higher proportions in aged mice, suggesting an impairment in immunomodulatory functions in these Foxp3^+^ cells (**Figures 9C**, lower right quadrant, **D**). More importantly, aged infected mice accrued pro-inflammatory effector CD4^+^ T cells expressing IFN-γ and IL-17A in oral mucosa even at later time points (day 30 after initial infection), indicating that the infection had led to the persistence of inflammatory CD4^+^ cells despite the absence of fungal burden in these mice (**Figures 8C**, **S11C**). To address the IL-1β/MyD88 mediated effects in Foxp3^+^ cells *in vivo*, we analyzed the expression of p-IRAK4 and IL-1R1 in Foxp3^+^ cells in oral mucosa (MOIL) derived from infected mice. Corroborating with lower IL-1β levels in aged mice (**Figure 8E**, top panel), Foxp3^+^ cells showed lower p-IRAK phosphorylation when compared to young mice (**Figure 9E**, left and right). These data suggest that IL-1β expressed during early time-points of infection may phosphorylate p-IRAK4 in IL-1R^+^T_regs_, which is critical for the activation and proliferation of these cells in oral mucosa. Furthermore, Foxp3^+^ cells from aged mice displayed lower levels of mTOR phosphorylation upon infection when compared to young mice (**Figure 9F**, top and bottom). In summary, these results indicate that excessive immunopathology in aged mice may be attributed to lower mTOR phosphorylation in Foxp3^+^ cells and diminished T_reg_17 cells during oral infection. These T_reg_17 defects also paralleled with impaired IL-1β induction, while excessive T_regDys_ accumulation could be attributed to elevated levels of IL-6 in oral mucosa of aged mice. These data are consistent with our *in vitro* results showing the contrasting roles of IL-6 and IL-1β on T_reg_ functions.

### Human Oral Mucosal Immune Dysfunction in Aged Individuals Parallels T_reg_ Alterations

**Figure 10:**
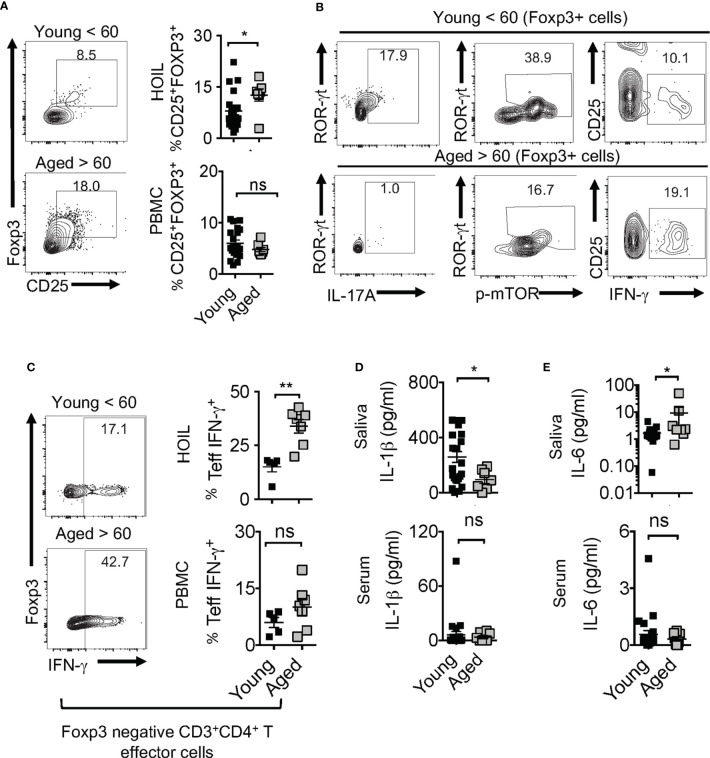
Aged individuals have decreased T_reg_17 cells and increased T_regDys_, correlating with CD4 T cell hyperactivation in oral mucosa. 32 human participants were recruited to the study (Male n=13, Female n=19; Aged < 60 n = 25; Aged > 60 n=7). Gingival biopsies and peripheral blood mononuclear cells (PBMC) were collected. Human oral intra-epithelial and lamina propria leukocytes (HOIL) and PBMC were obtained by processing gingival biopsy tissues and blood, respectively. Flow cytometric plots showing HOIL Foxp3 and CD25 expression; Geometric Mean of CD25; Young=1387 Aged= 744 **(A)** and statistical analysis of T_reg_ proportions in CD3^+^CD4^+^ of HOIL (above) and PBMC (below) (n=32). **(B)** Flow cytometric plots showing ROR-γt and IL-17A (left), ROR-γt, p-mTOR(middle), CD25, and IFN-γ (right) in HOIL Foxp3+ cells. **(C)** Flow cytometric analyses of non-T_reg_ CD4^+^ HOIL (above) and PBMC (below) cells showing IFN-γ expression *ex vivo* (n=12) (CD3^+^CD4^+^Foxp3^neg^ gated). ELISA quantification of IL-6 **(D)**, and IL-1β **(E)**, in saliva (above) and serum (below) collected from the participants. Mean values ± SEM are plotted. (*P < 0.05; Mann Whitney test). **P < 0.005. NS, non-significant.

Human aging is associated with systemic inflammation and oral manifestations associated with elevated IL-6 levels and immune dysfunction (54–56), although the underlying defects in immunomodulatory mechanisms are unclear. Based on the above results in aged mice, we turned to interrogate the physiological relevance of IL-1β/MyD88 defects and T_reg_ dysfunction in human oral mucosa of elderly individuals. To this end, we recruited 32 human participants that included young (age <60) and elderly individuals (age >60), and collected their saliva, peripheral blood mononuclear cells (PBMC) and oral gingival mucosa biopsies. We performed CD4 T cell immunophenotyping in human oral intra-epithelial and lamina propria leukocytes (HOIL) in gingival mucosa from aged and young groups (**Figure S12**). Although the overall CD4^+^ T cell proportions in HOIL were slightly lower (**Figure S12**, bottom), T_reg_ percentages were significantly higher in elderly individuals compared to the younger group (**Figure 10A**). However, T_reg_ proportions were comparable in PBMC of these groups, again showing a compartmentalized effect on mucosal T_regs_ (**Figure 10A**, bottom). We also observed a blunted expression of CD25 in these T_reg_ cells in aged individuals (**Figure 10A**, X-axis; see legend). Remarkably, examination of Foxp3^+^ cells showed that aged HOIL had significantly lower proportions of T_reg_17 cells compared to younger counterparts (**Figure 10B**, left). Aged Foxp3^+^ cells also displayed dampened mTOR phosphorylation in ROR-γt^+^ fraction (**Figure 10B** **, middle**), but higher proportions of T_regDys_ cells compared to young Foxp3^+^ cells **(** **Figure 10B**, right). Finally, gating on non-Foxp3 CD4^+^ effector cells, we found that CD4^+^ T cells from the elderly group showed a significantly higher frequency of IFN-γ expressing effector cells than younger individuals in HOIL (**Figure 10C**). This result suggested that oral mucosal CD4^+^ T cells in elderly individuals may have heightened activated state, despite an increase in T_regs_. Again, these alterations showed significant differences in HOIL, but not in PBMC (**Figure 10C**, right). These data suggested a dysregulated MyD88 signaling in T_regs_ due to IL-1β/IL-6 alterations in oral mucosa of aging humans, consistent to what we observed in aged mice. Validating this tenet, IL-1β levels were significantly diminished in saliva from aged individuals, although there were no differences observed in serum (**Figure 10D**). However, IL-6 was found to be elevated in aged individuals in saliva but not in serum (**Figure 10E**), implicating its role in expansion of oral mucosal T_regDys_ cells in aged group (**Figure 10C**). These data concur to the results from mouse experiments, which showed that dysregulation in IL-1β/IL-6 balance may contribute to oral T_reg_ dysfunction in aging mucosa.

## Discussion

Our results reveal a previously unknown role of the IL-1β/MyD88/mTOR axis in modulating mucosal T_regs_ during an infection. Although IL-1β is a conventional pro-inflammatory cytokine, our study unveils an unexpected and surprising role of this cytokine in inducing functionally robust Foxp3^+^ cells, namely T_reg_17 cells, during the early phases of infection. While TLR-2-MyD88 promotes Foxp3^+^ cell expansion, IL-1β produced by APC induces IL-17A expression and enhances the proliferation of these T_regs_. At the cellular level, we show that IL-1β/MyD88 mediated generation of T_reg_17 cells involves the conversion of both conventional CD4^+^ cells and Foxp3^+^T_reg_ cells into T_reg_17 cells in oral mucosa (**Figure S4**, **Figures 1**, **5**, and **6**). Using MyD88^-/-^ T_regs_ we show that T_reg_17 cells are critical for antifungal immunity and constraining inflammatory reactions to *Candida in vivo*. Loss of oral mucosal T_regs_ only in CLN and MOIL, but not in spleen of MFYcre mice (**Figure 1B**), signifies the compartmentalized effect of MyD88 signals through *Candida* and possibly also the local oral microbiome in mucosa (**Figure 1**). While our previous report demonstrated the role of short chain fatty acids (SCFA) in T_reg_ functions, (27, 31), our current study highlights the role of MyD88. Infection related immunopathology is unlikely a consequence of hyperactivated CD4 cells present in MFYcre mice before infection (**Figure S2**). It is rather a result of T_reg_ specific defects during infection, because the effector CD4 cells in sham mice had comparable levels of cytokines in both FYcre and MFYcre mice (**Figures 3A, B**). However, after the infection, MFYcre mice exhibit excessive proportions of Ly6C^high^ macrophages and Gr-1+ neutrophils, as well as elevated IFN-γ expression in effector CD4+ cells (**Figures 3F–H**). These suggest infection related immunopathology. While we speculate that excessive CD4^+^IFN-γ may contribute to the persistence of pro-inflammatory monocytes producing IL-6, this possibility remains to be evaluated in the future.

On the one hand, T_regs_ are known to enhance Th17 mediated host immunity and fungal clearance at early time-points during oral *Candida* infection (28). Consistently, absence of MyD88 in T_regs_ resulted in diminution of T_regs_, decreased Th17 cells, and increased fungal burden (**Figures 2C**, **3A, B**). Therefore, MyD88 is required in T_regs_ for their basal proliferation and enhancing Th17 functions at early time-points of infection. On the other, T_regs_ also reduce Gr-1+ (Ly6G) neutrophils and tongue inflammation at later time-points of infection (21). Concurrently, CD4^+^ cells with little or no regulation by T_regs_ contributed to hyperactivated CD4 T cells, inflammatory monocytes, neutrophils, and tongue immunopathology in *Candida* infected MFYcre mice (**Figures 2D**, **3C**, **4**). Increased infiltration and persistence of these cells at late-time points led us to infer that these mice suffer immunopathology. While a non-resolving infection could also have resulted in increased infiltration of these cells in MFYcre mice, aging mice did not have increased fungal burden but still displayed tongue pathology. Although IL-1β/MyD88 deficiency in T_regs_ is a common link in poor infection outcomes in our MFYcre and aging infection models, there are distinct differences between them. Increased fungal burden and immunopathology are driven predominantly by reduced T_reg_/T_reg_17 numbers and functions in MFYcre mice. Moreover, loss of T_regs_ paralleled with the loss of Th17 cells in these mice. Therefore, immunopathology was driven by reduced antifungal immunity and also impaired ability of T_regs_ to control inflammation. However, aged mice showed increased tongue immunopathology even with an intact ability to clear the fungus. These data are congruent with our results that aged mice have intact Foxp3^+^ cells and intact Th17 immunity. While mechanisms are unclear, it is tempting to speculate that basal hyperinflammatory state in aging mice might additionally contribute to some of these differences. Higher expression of IFN-γ observed in SPLN, even in the absence of infection, indicates such a hyperinflammatory state in aged mice (**Figure 9D**). Taken together, these results led us to infer that reduced T_reg_17 cells and increased dysfunctional T_regs_, but not increased fungal burden alone, contributed to immunopathology outcome during *Candida* infection.

In line with previous studies, the mechanism of T_reg_ functions may involve their ability to consume IL-2 leading to the regulation of IL-2 receptor (CD25) (**Figures 4C, D**) and IFN-γ in CD4^+^ cells [**Figures 3C**, **9A** (21, 26, 28, 57–59)]. Our current data support the possibility that in the context of early IL-1β expression by APC, T_reg_/T_reg_17 mediated IL-2 regulation enhance Th17 cells at early phases. At later phases of infection, T_reg_17 mediated IL-2 consumption and increased TGF-β1 expression in T_regs_ may downmodulate inflammation (21, 28). However, excessive IL-6 may dysregulate this mechanism of IL-2 consumption mediated immunoregulation by T_regs_. Supporting this tenet, reduced CD25 expression in aged Foxp3^+^ cells (**Figure 8D**, X-axis, **Figure 10A**, X-axis) and their likely inability to regulate IL-2 is likely associated with their dysfunction and immunopathology during infection in aged mice (26, 57–59). These data are also in accord with previous reports showing an association of CD25^low^ Foxp3^+^ cells with tissue autoimmunity (60). The contribution of IL-17A in the immunomodulatory role of T_reg_17 cells is currently unclear. We cannot rule out the function of this cytokine working in concert with immunomodulatory DC or macrophages unique to the mucosal environment during *Candida* infection (61, 62). While these T_reg_17 cells appear to be consistent with IL-1R1^+^CD25^+^ tissue T_reg_ phenotype (63), other molecular features that are associated with their regulatory function *in vivo* remain to be investigated. Absence of differences in IL-10 expression among Foxp3^+^ cells between FYcre and MFYcre groups (**Figure S6A**) led us to infer that IL-10 might not be involved in T_reg_17 mediated immunomodulation in our system.

In light of the current findings we postulate that *Candida* induces mature IL-1β expression in APC which in the context of synergistic IL-1R/TGF-β1 signaling, promotes T_reg_17 cells. Expression of TLR-2, IL-1R, as well as IL-17A in tT_regs_ and pT_regs,_ verifies this postulate (2). At the molecular level, IL-1β induces the activation of IRAK-4, Akt, mTOR, and p70-S6K axis in naïve CD4^+^ T cells and Foxp3^+^T_regs_ in an IL-1R dependent manner (**Figure 7**). mTOR activation in T_regs_ is crucial for effector T_reg_ function *in vivo* (30, 64). Concurringly, here we show that MyD88/IL-1β mediated mTOR activation induces T_reg_17 cells *in vitro* and *in vivo* (**Figures 2**, **7**, **10**). Surprisingly, our current study also revealed that IL-1β restrains IL-6 dependent IFN-γ expression in Foxp3^+^ cells during *Candida* activation (**Figure 6E**). Blocking IL-6 did not alter T_reg_17 induction in IL-1R^-/-^ cells, which suggests that IL-6 acts synergistically with IL-1β and cannot independently induce IL-17A in Foxp3^+^ cells. However, the mechanism by which IL-1β interacts with IL-6 signaling and constrains IFN-γ expression in Foxp3^+^ cells is currently unclear and remains to be studied. Also, the identity of oral mucosal monocytes or macrophages that express active IL-1β/IL-6 during an infection is unknown and warrants future studies (49).

Previous reports show that IFN-γ secretion by Foxp3^+^ cells is associated with T_reg_ dysfunction (38, 65). Consistently, our study shows that in the context of excessive IL-6, IL-1β/MyD88 deficiency in Foxp3^+^ cells contributes to their IFN-γ secretion. Such IFN-γ expressing T_regDys_ cells fail to control oral mucosal inflammation and host defense. While IL-6 is required for Th17 development and resistance to *Candida* infection (66), our data show that elevated levels of IL-6 are strongly associated with Foxp3^+^ T_regDys_ phenotype and immunopathology in infected aged mice (**Figure 9**). These data are also concurrent with elevated IL-6 observed in patients with chronic mucocutaneous candidiasis (67). Our data also imply that elevated IL-6 and dysbiosis, which might include *Candida* dysbiosis, may contribute to T_reg_ dysfunction and CD4 hyperactivation in aging human oral mucosa (**Figure 10**) (68). Thus, our study supports that IL-1β/IL-6 imbalance is central to Foxp3^+^ cell plasticity and inflammation control during *Candida* infection (**Figures 8E–H**, **9A**). Although we did not evaluate the effect of microbial dysbiosis in T_reg_ dysfunction in elderly participants, we observed elevated IL-6 levels and CD4^+^ T cell dysfunction in aging human individuals (**Figure 10**). These data may partially explain the higher prevalence and mortality of *Candida* infections in elderly patients and warrant future studies examining T_reg_17 cells during *Candida* infection. Taken together, our study reveals a central role of IL-1β-mTOR-T_reg_17 axis in controlling oral inflammation and provides an insight into how dysregulation of this mechanism could contribute to overt inflammation during mucosal infections in elderly individuals. It also suggests that manipulating this signaling represents a potential strategy to target T_reg_ functions in mucosa.

## Data Availability Statement

The original contributions presented in the study are included in the article/**Supplementary Material**. Further inquiries can be directed to the corresponding author.

## Ethics Statement

The studies involving human participants were reviewed and approved by IRB, University Hospitals, CWRU. The patients/participants provided their written informed consent to participate in this study. The animal study was reviewed and approved by IACUC.

## Author Contributions

PP designed the study, performed experiments, analyzed data, supervised the project, and wrote the manuscript. FF and AP provided gingival biopsies from human participants. NB and ES performed the experiments, genotyping, and analyzed ELISA and qPCR data, and contributed to discussions. PM performed validation qPCRs and analyzed the data. ES scored the infected mice in a blinded fashion and isolated mouse tissues. *Foxp3 YFP^cre^* and *Myd88^flox/flox^* mice were bred in NIAID, NIH mouse facility before transferring to the mouse facility at CWRU. SJ performed microscopy of the histology slides and analyzed the data. AW read the manuscript and contributed to discussions. All authors contributed to the article and approved the submitted version.

## Funding

PP was supported by departmental startup funding from CWRU School of Dental Medicine, CWRU Skin disease research center pilot funding P30AR039750-19, CWRU Center for AIDS Research (CFAR) Catalytic award, and RO1DE026923 NIH/NIDCR funding. 

## Conflict of Interest

The authors declare that the research was conducted in the absence of any commercial or financial relationships that could be construed as a potential conflict of interest.

## Abbreviations

CA, *Candida albicans*; CLN, Cervical lymph nodes; FYcre, Foxp3^YFPcre^ mice; HKGT, Heat killed *Candida albicans* germ tube; HOIL, Human oral intra-epithelial and lamina propria leukocytes; MFYcre, Mice with Foxp3^+^ cell specific deletion of MyD88; MyD88 crossed with Foxp3^YFPcre^; MyD88, Myeloid differentiation primary response 88; MOIL, Mouse oral intra-epithelial and lamina propria leukocytes; OPC, Oropharyngeal candidiasis; SPLN, Spleen; T_reg_IFN-γ, IFN-γ expressing dysfunctional Foxp3^+^ cells; T_reg_17, IL-17A expressing Foxp3^+^ cells.
