# Supplementary material for: IL-1β-MyD88-mTOR Axis Promotes Immune-Protective IL-17A+Foxp3+ Cells During Mucosal Infection and Is Dysregulated With Aging
Source: Front Immunol. 2020 Nov 6;11:595936. doi: 10.3389/fimmu.2020.595936 (PMC7677307; doi:10.3389/fimmu.2020.595936)

## Slide 1
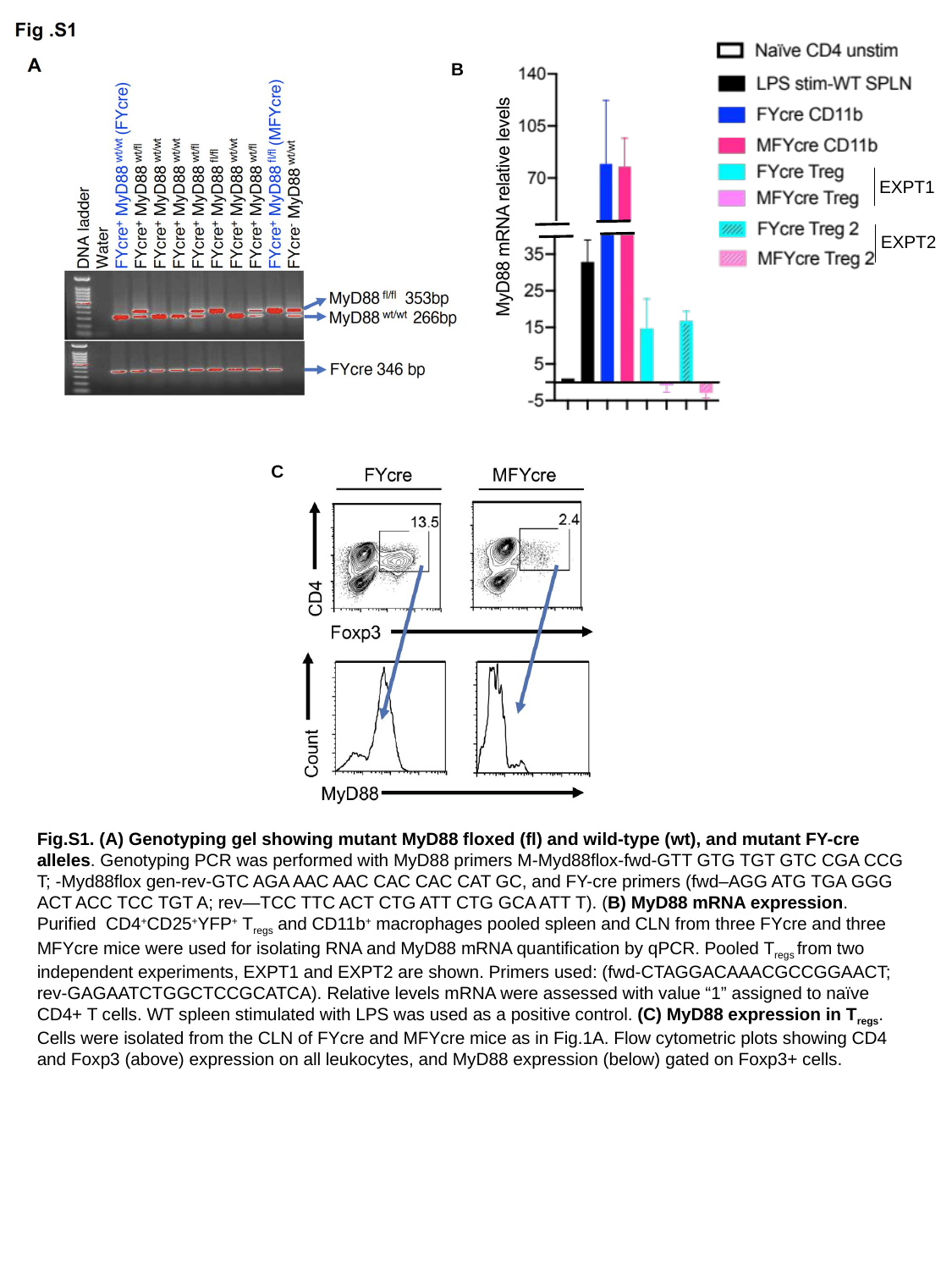

B
EXPT1
EXPT2
C
Fig.S1. (A) Genotyping gel showing mutant MyD88 floxed (fl) and wild-type (wt), and mutant FY-cre
alleles. Genotyping PCR was performed with MyD88 primers M-Myd88flox-fwd-GTT GTG TGT GTC CGA CCG
T; -Myd88flox gen-rev-GTC AGA AAC AAC CAC CAC CAT GC, and FY-cre primers (fwd–AGG ATG TGA GGG
ACT ACC TCC TGT A; rev—TCC TTC ACT CTG ATT CTG GCA ATT T). (B) MyD88 mRNA expression. Purified CD4+CD25+YFP+ Tregs and CD11b+ macrophages pooled spleen and CLN from three FYcre and three MFYcre mice were used for isolating RNA and MyD88 mRNA quantification by qPCR. Pooled Tregs from two independent experiments, EXPT1 and EXPT2 are shown. Primers used: (fwd-CTAGGACAAACGCCGGAACT; rev-GAGAATCTGGCTCCGCATCA). Relative levels mRNA were assessed with value “1” assigned to naïve CD4+ T cells. WT spleen stimulated with LPS was used as a positive control. (C) MyD88 expression in Tregs. Cells were isolated from the CLN of FYcre and MFYcre mice as in Fig.1A. Flow cytometric plots showing CD4 and Foxp3 (above) expression on all leukocytes, and MyD88 expression (below) gated on Foxp3+ cells.

## Slide 2
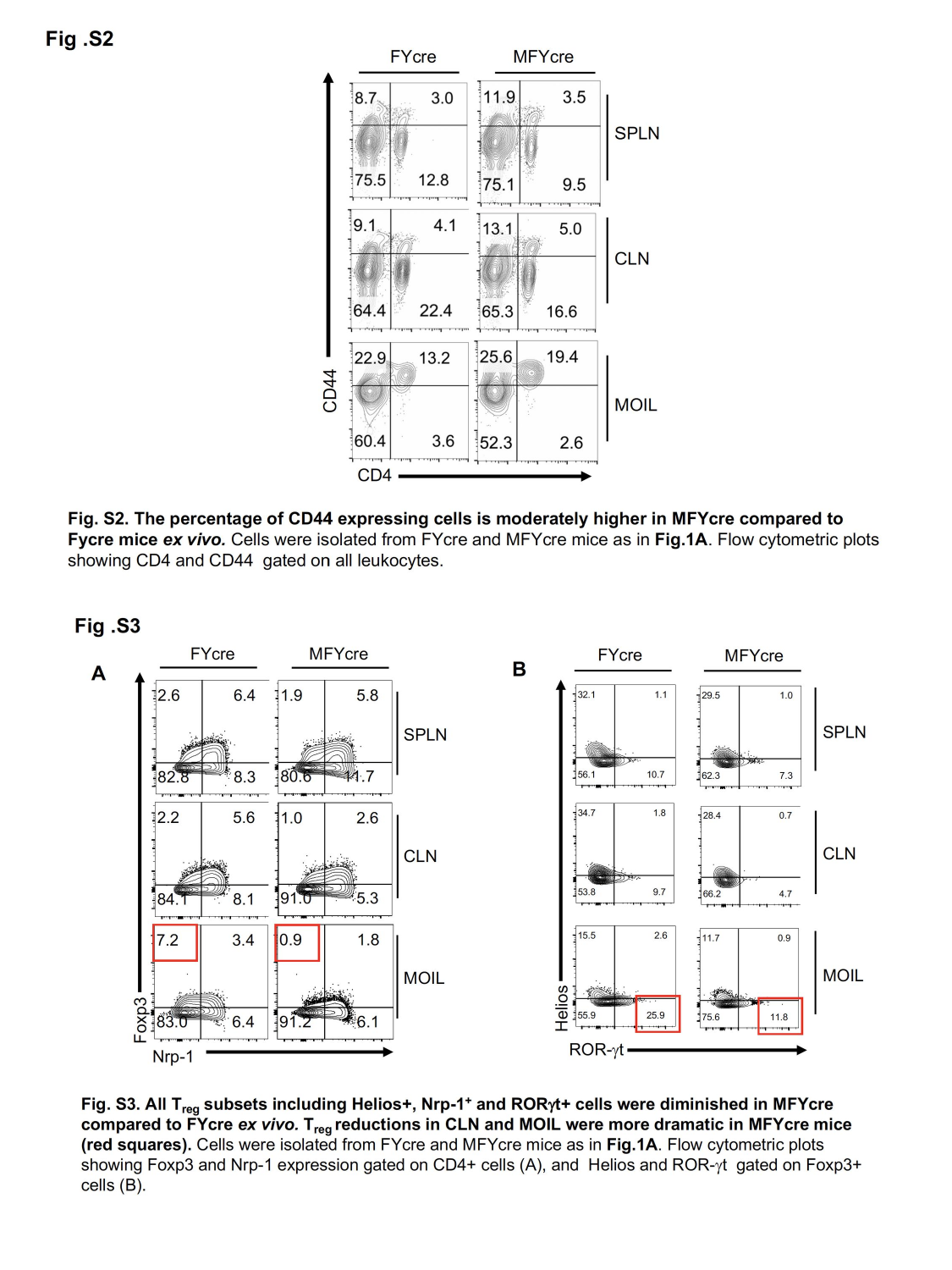

## Slide 3
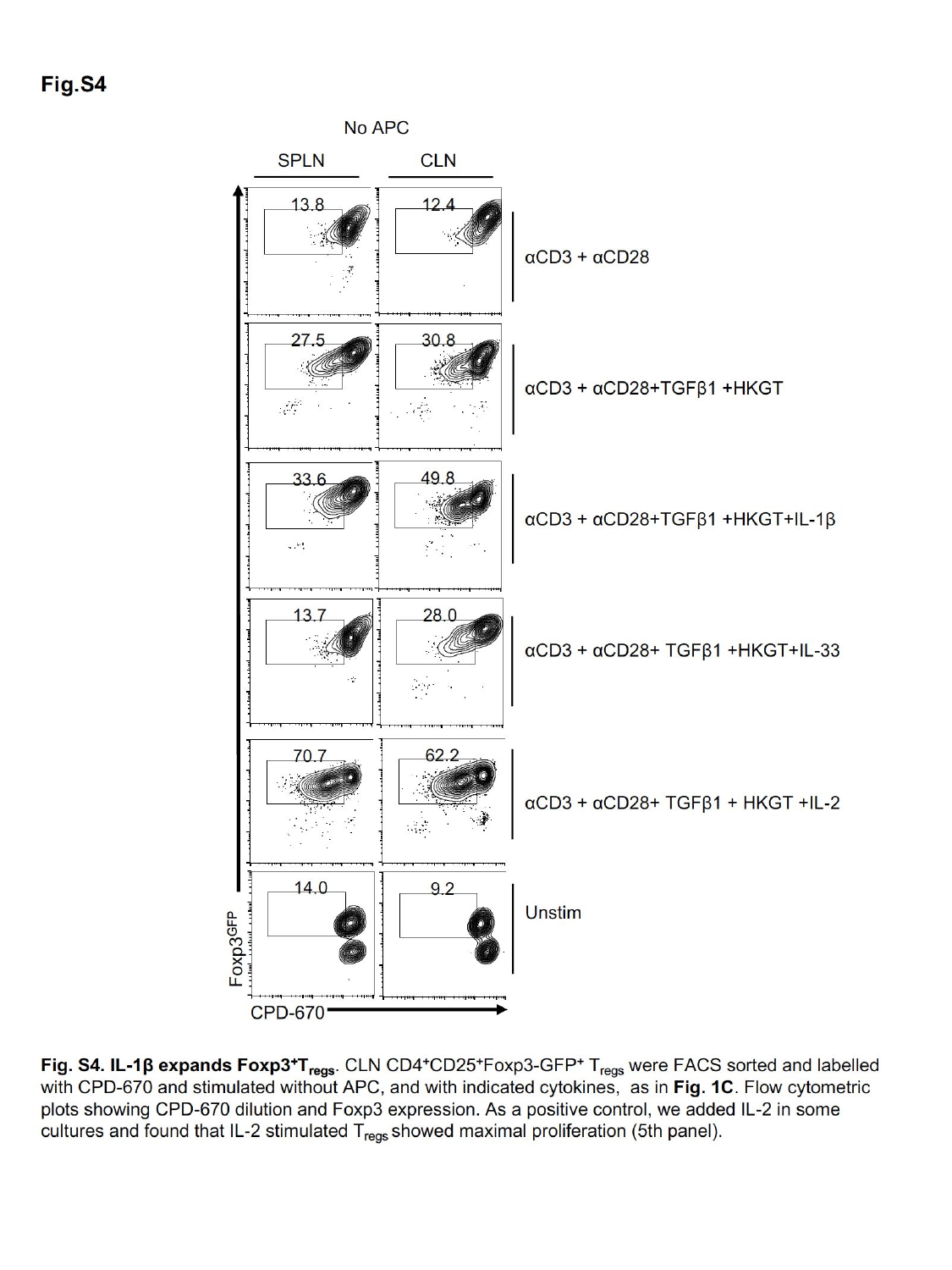

#

## Slide 4
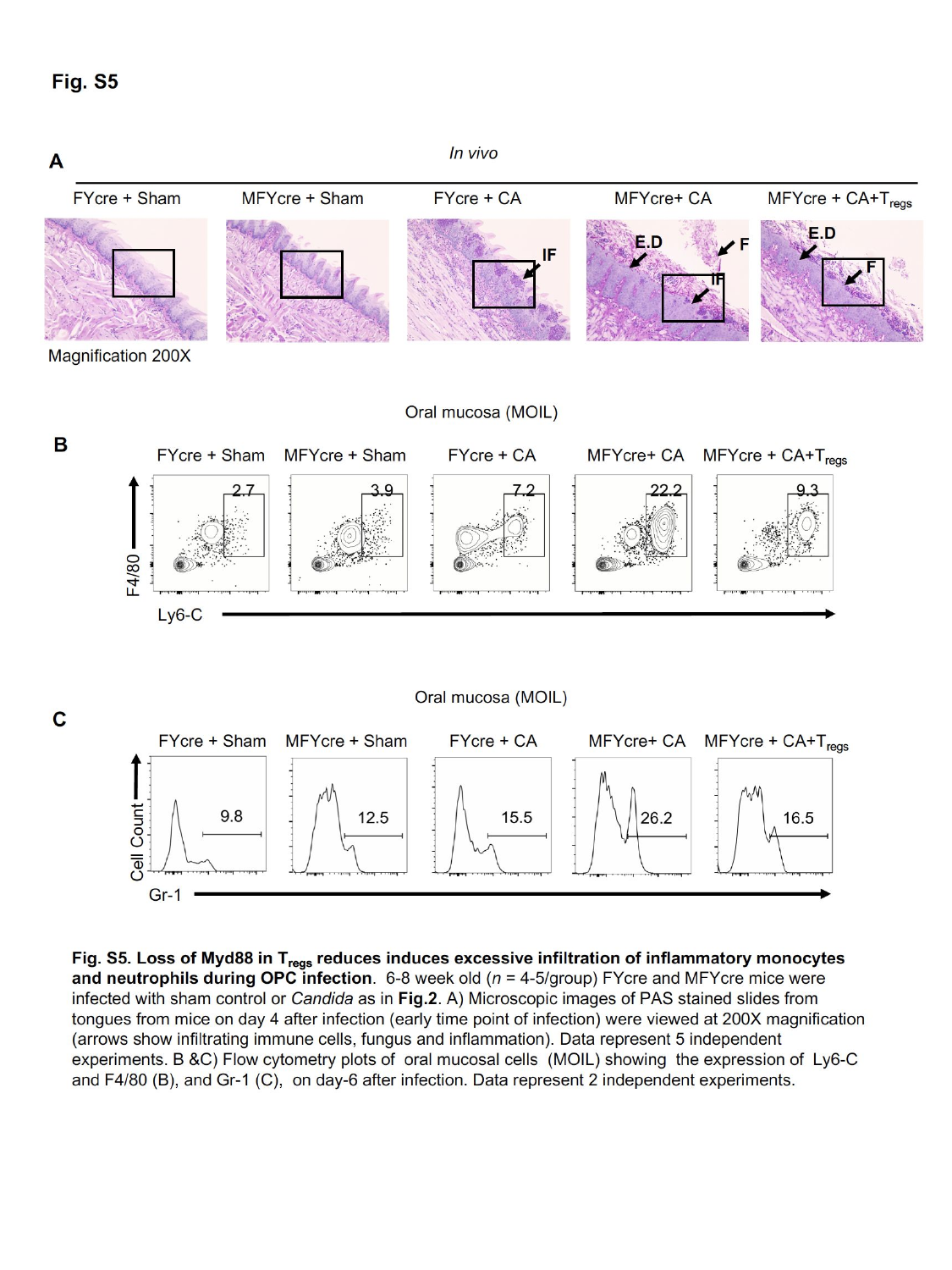

## Slide 5
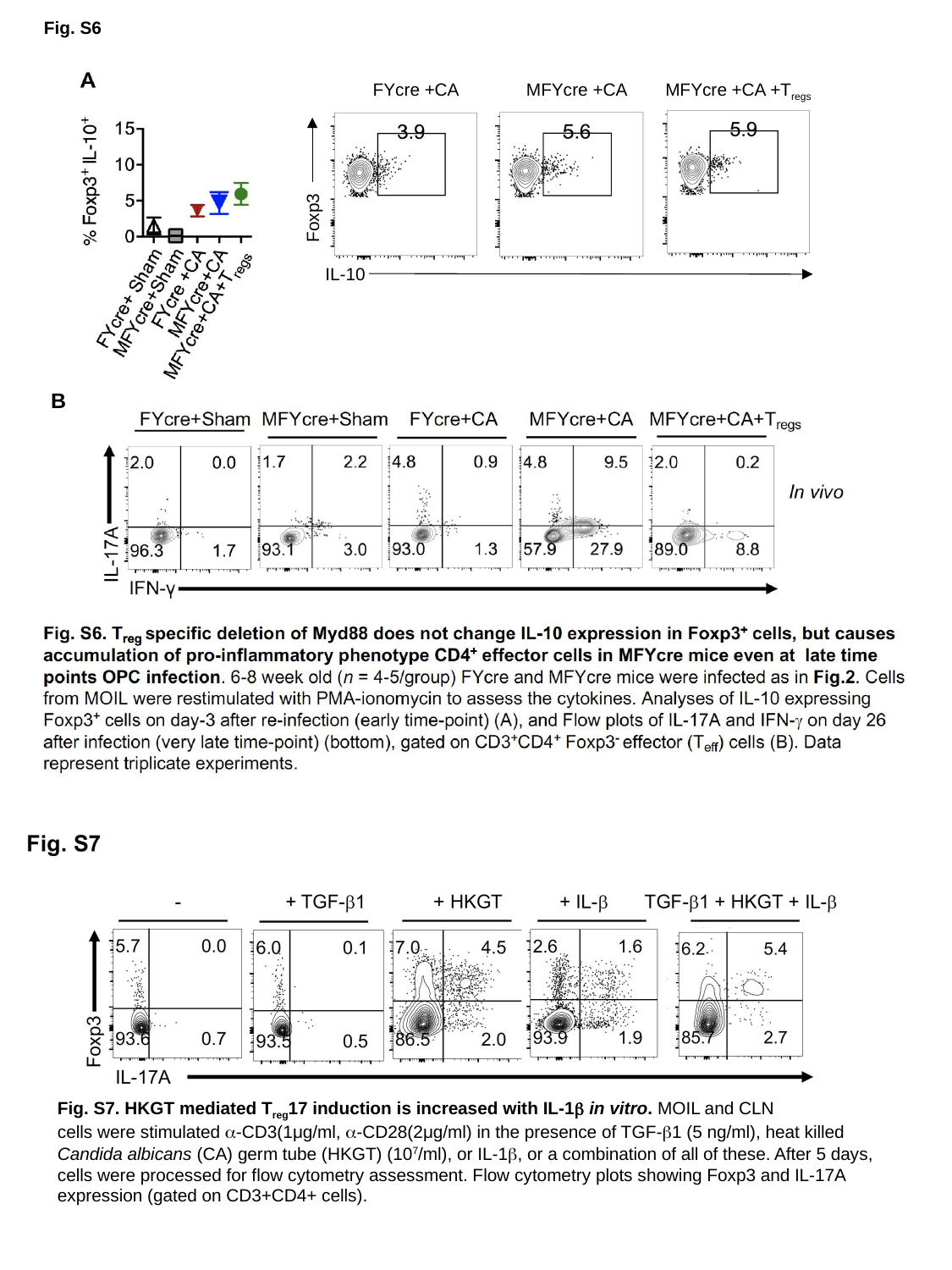

Fig. S6
FYcre +CA MFYcre +CA MFYcre +CA +Tregs
Foxp3
IL-10
B
Fig. S7. HKGT mediated Treg17 induction is increased with IL-1b in vitro. MOIL and CLN
cells were stimulated a-CD3(1μg/ml, a-CD28(2μg/ml) in the presence of TGF-b1 (5 ng/ml), heat killed
Candida albicans (CA) germ tube (HKGT) (107/ml), or IL-1b, or a combination of all of these. After 5 days,
cells were processed for flow cytometry assessment. Flow cytometry plots showing Foxp3 and IL-17A
expression (gated on CD3+CD4+ cells).

## Slide 6
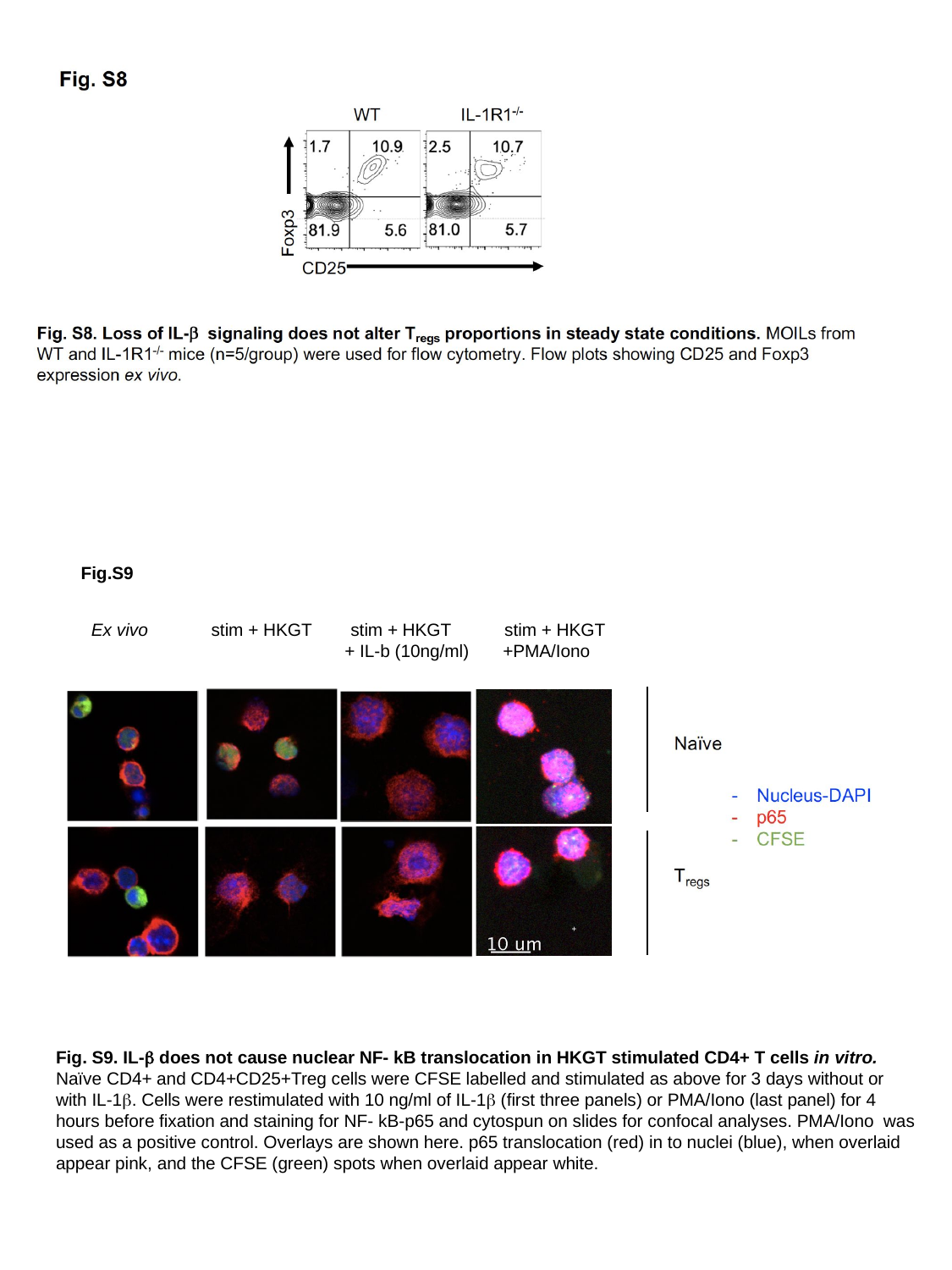

Fig.S9
Ex vivo stim + HKGT stim + HKGT stim + HKGT
	 + IL-b (10ng/ml) +PMA/Iono
Fig. S9. IL-b does not cause nuclear NF- kB translocation in HKGT stimulated CD4+ T cells in vitro.
Naïve CD4+ and CD4+CD25+Treg cells were CFSE labelled and stimulated as above for 3 days without or
with IL-1b. Cells were restimulated with 10 ng/ml of IL-1b (first three panels) or PMA/Iono (last panel) for 4 hours before fixation and staining for NF- kB-p65 and cytospun on slides for confocal analyses. PMA/Iono was used as a positive control. Overlays are shown here. p65 translocation (red) in to nuclei (blue), when overlaid appear pink, and the CFSE (green) spots when overlaid appear white.

## Slide 7
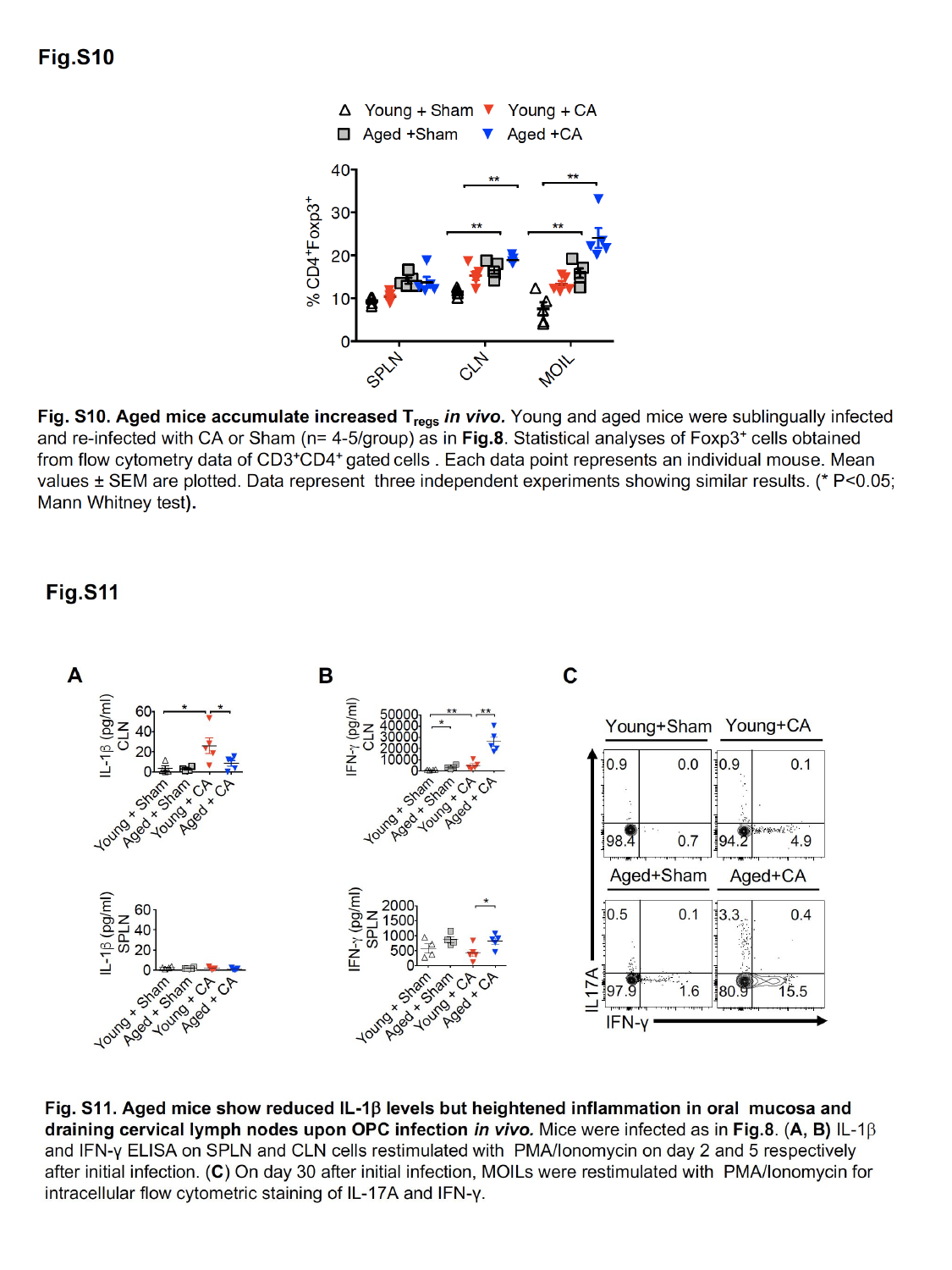

## Slide 8
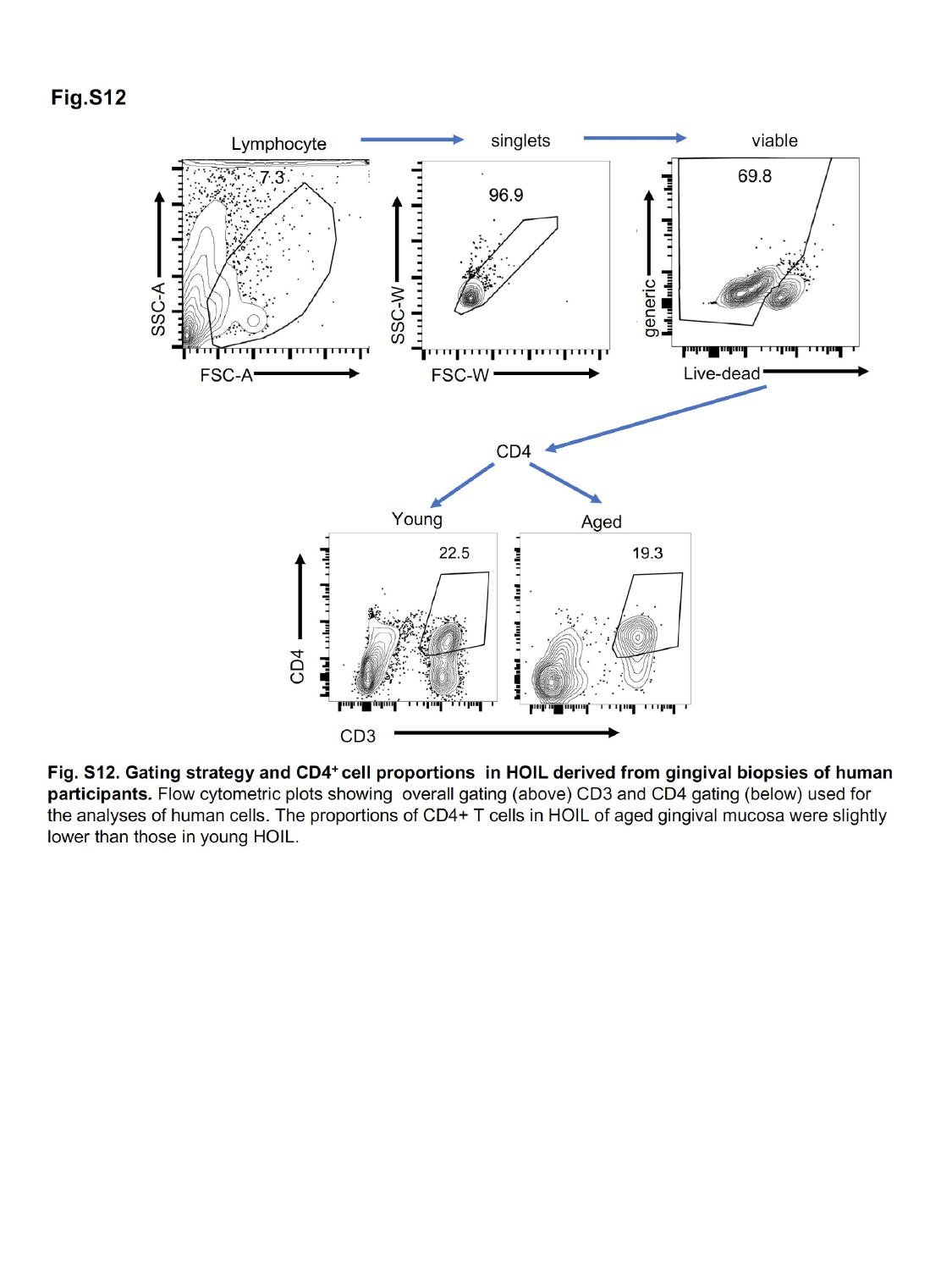

Supplement: Supplementary file 1 [file Presentation_1.pptx]
